# The Osteology of the Basal Archosauromorph *Tasmaniosaurus triassicus* from the Lower Triassic of Tasmania, Australia

**DOI:** 10.1371/journal.pone.0086864

**Published:** 2014-01-30

**Authors:** Martín D. Ezcurra

**Affiliations:** 1 School of Geography, Earth and Environmental Sciences, University of Birmingham, Birmingham, United Kingdom; 2 GeoBio-Center, Ludwig-Maximilian-Universität München, Munich, Germany; Ghent University, Belgium

## Abstract

Proterosuchidae are the most taxonomically diverse archosauromorph reptiles sampled in the immediate aftermath of the Permo-Triassic mass extinction and represent the earliest radiation of Archosauriformes (archosaurs and closely related species). Proterosuchids are potentially represented by approximately 15 nominal species collected from South Africa, China, Russia, Australia and India, but the taxonomic content of the group is currently in a state of flux because of the poor anatomic and systematic information available for several of its putative members. Here, the putative proterosuchid *Tasmaniosaurus triassicus* from the Lower Triassic of Hobart, Tasmania (Australia), is redescribed. The holotype and currently only known specimen includes cranial and postcranial remains and the revision of this material sheds new light on the anatomy of the animal, including new data on the cranial endocast. Several bones are re-identified or reinterpreted, contrasting with the descriptions of previous authors. The new information provided here shows that *Tasmaniosaurus* closely resembles the South African proterosuchid *Proterosuchus*, but it differed in the presence of, for example, a slightly downturned premaxilla, a shorter anterior process of maxilla, and a diamond-shaped anterior end of interclavicle. Previous claims for the presence of gut contents in the holotype of *Tasmaniosaurus* are considered ambiguous. The description of the cranial endocast of *Tasmaniosaurus* provides for the first time information about the anatomy of this region in proterosuchids. The cranial endocast preserves possibly part of the vomero-nasal ( = Jacobson’s) system laterally to the olfactory bulbs. Previous claims of the absence of the vomero-nasal organs in archosaurs, which is suggested by the extant phylogenetic bracket, are questioned because its absence in both clades of extant archosaurs seems to be directly related with the independent acquisition of a non-ground living mode of life.

## Introduction

Archosauromorpha is a major group within diapsid reptiles that includes living birds and crocodilians, as well as all extinct species more closely related to these living groups than to lepidosaurs (lizards, snakes and *Sphenodon*) [1]. The oldest known archosauromorphs are from Upper Permian rocks of Europe [2]–[4], Russia [5]–[7] and Tanzania [8]. However, it is not until the aftermath of the Permo-Triassic mass extinction (which occurred at ca. 252.6 Ma [9]) that the archosauromorph fossil record documents the presence of morphologically diverse and taxonomically abundant groups, including members of Rhynchosauria, Prolacertiformes, Proterosuchidae and Archosauria [10]–[16]. Proterosuchidae constitutes the most species rich clade of archosauromorphs sampled during the biotic recovery that took place during the Early Triassic and early Middle Triassic and represents the earliest radiation of Archosauriformes, the group that includes crown archosaurs such as crocodiles and dinosaurs, as well as multiple non-crown groups that disappeared before or at the Triassic–Jurassic boundary [16]–[18]. Proterosuchidae is potentially represented by approximately 15 nominal species collected from South Africa, China, Russia, Australia and India, but the taxonomic content of the group is currently in a state of flux because of the poor knowledge of the anatomy, taxonomic status and systematic position of several of its members [16]. One of the putative proterosuchid species that deserves restudy is *Tasmaniosaurus triassicus* from the Lower Triassic of Tasmania, Australia. This species is known from a single partial skeleton, including cranial and postcranial remains that were originally described by Camp & Banks [19]. Subsequently, Thulborn [20] provided multiple re-interpretations of the anatomy of *Tasmaniosaurus triassicus* and reported in this species the first gut contents known for a proterosuchid. A first hand re-study of *Tasmaniosaurus triassicus* was conducted due to its importance for understanding the anatomy, systematics and palaeobiology of early archosauriforms. The new information gathered from this re-examination provides novel data on the anatomy of the species, including severeal re-interpretations (Table 1), that will contribute to future clarifications of the taxonomy and systematics of Proterosuchidae and other early Archosauriformes.

**Table 1:** Comparison of previous identifications of bones of the holotype of *Tasmaniosaurus triassicus* (UTGD 54655) and those of the present paper.

Camp & Banks (1978) [19]	Thulborn (1986) [20]	This paper
left premaxilla	right premaxilla	right premaxilla
right premaxilla (anterior)	?right dentary+? left maxilla	left dentary (part)
right premaxilla (posterior)	?maxilla	right maxilla
right maxilla	right maxilla	?left maxilla
left maxilla	?maxilla or ?dentary	left dentary (part)
frontals	nasals	frontals
right postorbital	rib	rib
left quadratojugal	?gastralia	right lacrimal
parietals	frontals+postorbitals	parietals
postfrontals	?prefrontals	postfrontals
interparietal	not located	interparietal
supraoccipital	?rib fragment	?supraoccipital
?left squamosal	?gastralia	indeterminate
right epipterygoid	indeterminate	?epipterygoid
?right pterygoid	indeterminate	indeterminate
ectopterygoid/vomer/pterygoid	indeterminate	right pterygoid
left dentary	left dentary+splenial	right dentary
left splenial	right dentary	left splenial
not located	left postorbital	right parietal (part)
not located	?right postorbital	indeterminate
not located	?left squamosal	indeterminate
not located	?parietal(s)	rib
dorsal vertebrae	dorsal vertebrae	cervico-dorsal vertebrae
caudal vertebrae	caudal vertebrae	caudal vertebrae
ribs	ribs	ribs
gastralia	gastralia	gastralia
haemal arches	haemal arches	haemal arches
interclavicle	interclavicle	interclavicle
?scapula	indeterminate	indeterminate
?ilium	?caudal vertebrae	caudal vertebrae
?pubis	indeterminate	indeterminate
?ischium	not located	not located
left tibia and fibula	limb bones	tibia and ?rib
right tibia	?left tibia	tibia
right fibula	?left femur	?femur
tarsal bones	indeterminate	indeterminate
pedal bones	?manual bones	pedal bones
pedal bones	pedal bones	pedal bones
not located	?left fibula	indeterminate

### Geological and Palaeontological Setting

**Figure 1:**
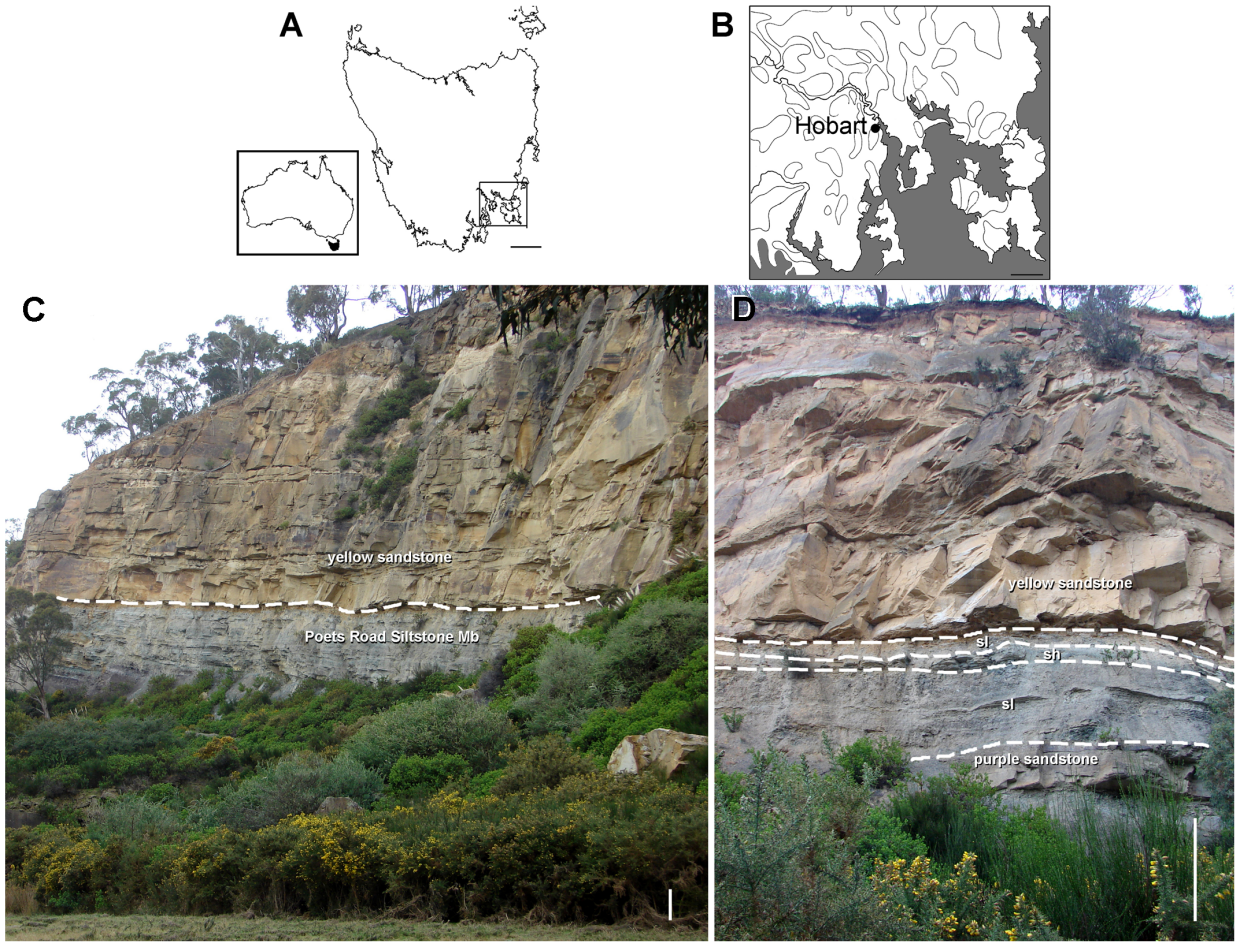
Geographic and stratigraphic occurrence of *Tasmaniosaurus triassicus*. **A**, map of Australia (small rectangle) and Tasmania showing the aera of Hobart in the rectangle; **B**, area of Hobart depicting the location of the city of Hobart; and **C**, **D**, photographs of the outcrop of the upper levels of the Poets Road Siltstone Member of the Knocklofty Formation and the overlying massive yellow sandstone. Abbreviations: sh, probable *Tasmaniosaurus*-bearing shale; sl, grey siltstone. Scale bars equal 50 km (A), 10 km (B), and 1 m (C, D).

In September 1960 John A. Townrow and Maxwell R. Banks found the remains of a small reptile in the Lower Triassic rocks that crop out in the Crisp and Gunn’s Quarry, in the western suburbs of Hobart, Tasmania (Fig. 1). The bones lay in a loose block of hard, light to medium grey shale that had fallen from the cliff face about nine metres above the base of the quarry, and the remains were collected in several smaller blocks detached from the upper side of this larger fallen block [19]. Although all the smaller blocks seem to correspond to the same area of the larger fallen block, the original arrangement of the different smaller pieces relative to one another is unknown (Banks pers. comm. 2012). The remains consist of a partial skeleton including cranial and postcranial bones, which were described by Camp & Banks [19] as the new genus and species *Tasmaniosaurus triassicus*. Camp & Banks [19] provided a detailed account of the geology of the Crisp and Gunn’s Quarry and correlated outcrops in the area, erecting the Poets Road Siltstone Member of the Knocklofty Formation (for a detailed description of the geology of the Poets Road Siltstone Member and the Crisp and Gunn’s Quarry see Camp & Banks [19]). These authors reported that the holotype of *Tasmaniosaurus triassicus* occurred in a coarse-grained well-sorted siltstone of about 10 centimetres thickness in the upper Poets Road Siltstone Member. A visit to the type locality of *Tasmaniosaurus triassicus* by the author in August 2012, following the original geological description of Camp & Banks [19], allowed the re-location of the siltstone level that probably yielded the type specimen (Banks pers. comm. 2012) (Fig. 1C, D). The grey siltstone (Fig. 1: sl) and its underlying purple siltstone with sandstone (Fig. 1D: purple sandstone), which is currently largely covered by fallen debris from higher levels of the quarry, correspond to the Poets Road Siltstone Member of the Knocklofty Formation (Fig. 1C). This member is overlain by a massive, approximately 25 metres thick package of yellow-light brown sandstone (Fig. 1C: yellow sandstone). The overall aspect of the outcrop is similar to that in 1960 (Banks pers. comm. 2012) but the base of the quarry is approximately 3 metres higher than when *Tasmaniosaurus triassicus* was discovered because of refill by fallen debris covering the lower levels of the Poets Road Siltstone Member (Fig. 1C, D). As a result, the level that probably yielded the *Tasmaniosaurus triassicus* remains is currently approximately 6 metres from the base of the quarry (Fig. 1D: sh). Thulborn [20] provided georeferenced coordinates in degrees and minutes (42° 53′ S 147°19’ E) for the whereabouts of the Crisp and Gunn’s Quarry and directed readers to the original map of Camp & Banks [19] for a more precise location. A recent visit to the type locality of *Tasmaniosaurus triassicus* allowed the collection of more precise coordinates: 42°52′50.0″ S 147°18′10.6″ E ±100 metres (a GPS was used to take the coordinates using the WGS-84 datum).

The Knocklofty Formation belongs to the upper levels of the Upper Parmeener Supergroup [21] and preserves a continental, fresh-water fossil assemblage that includes *Tasmaniosaurus triassicus*, as well as five nominal species of temnospondyls [22] and several genera of osteichthyan fishes [23]. In particular, the Crisp and Gunn’s Quarry has yielded fossil tetrapods at several stratigraphic levels, including the temnospondyls *Chomatobatrachus halei* and *Rotaurisaurus contundo* at the very base of the quarry floor of 1960, which is currently at least partially covered by fallen debris, and *Banksiops townrowi* ( =  “*Blinasaurus townrowi*”) in the massive yellow sandstone that overlies the Poets Road Siltstone Member [22], [24]. The grey, *Tasmaniosaurus*-bearing siltstone in the upper Poets Road Siltstone Member at the Crisp and Gunn’s Quarry has also yielded conchostracan, fish and temnospondyl fossil remains [19]. Camp & Banks [19] interpreted the depositional environment of the *Tasmaniosaurus*-bearing level as a pond or small lake.

The Knocklofty Formation has been traditionally interpreted to be Early Triassic in age based on palynomorphs [25], vertebrate biostratigraphy [19], [22] and overall similarities with other Early Triassic vertebrate communities based on numerical analyses [26]. Palynomorphs suggest a Griesbachian (early Induan) or Nammalian (late Induan–early Olenekian) age [25]. The osteichthyan species are not useful for biostratigraphic correlations due to their long biochrons [23] and the position of *Tasmaniosaurus triassicus* among archosauromorphs has not yet been tested in a quantitative phylogenetic analysis [16]. As a result, vertebrate biostratigraphy has been mostly restricted to temnospondyl occurrences [22]. The Knocklofty Formation has yielded the following temnospondyl species: the brachyopid *Banksiops townrowi*, the lydekkerinid *Chomatobatrachus halei*, the lapillopsid *Rotaurisaurus contundo* and the rhytidosteids *Deltasaurus kimberleyensis* and *Derwentia warreni* [22], [24].

Warren & Marsicano [27] found the brachyopid *Banksiops townrowi* as the sister-taxon of *Batrachosuchus browni* and *Batrachosuchus watsoni*, both probably from the *Cynognathus* Assemblage Zone (AZ) Sub-Zones A and B of South Africa, although there is uncertainty regarding their exact stratigraphic ocurrences [28]. Damiani [29] recovered *Chomatobatrachus halei* in a polytomy together with the other lydekkerinids *Watsonisuchus madagascariensis, Deltacephalus whitei* and *Lydekkerina huxleyi.* Subsequently, Steyer [30] removed *Watsonisuchus madagascariensis* from the lydekkerinids and recovered it alternatively as a mastodonsaurid. *Lydekkerina huxleyi* is found through the entire *Lystrosaurus* AZ of South Africa [28] and coeval levels of the Rewan Formation of Australia [31]. *Deltacephalus whitei* is from the Middle Sakamena Formation of Madagascar, which has been correlated with the *Lystrosaurus* AZ of South Africa based on temnospondyl biostratigraphy [32]. *Rotaurisaurus contundo* was found as the sister-taxon of *Lapillopsis nana* from the Lower Triassic lower Arcadia Formation that crops out in northeastern Australia [33]. *Deltasaurus kimberleyensis* and *Derwentia warrenii* are included within the family Rhytidosteidae, which has a single record in the latest Permian but most of its members are restricted to the Early Triassic [34]. Accordingly, temnospondyls suggest a correlation of the Knocklofty Formation with the *Lystrosaurus* AZ of South Africa (Induan–early Olenekian) [35], which is in agreement with the evidence provided by palynomorphs. Recent analysis of detrital zircons from the lower levels of the Upper Parmeener Supergroup yielded a maximum depositional age of 253±4 million years [36], ranging from the early Wuchiapingian to the middle Olenekian (sensu Gradstein et al. [9]). As a result, this absolute dating indicates a maximum depositional age for the Knocklofty Formation that is in agreement with biostratigraphical correlations.

## Materials and Methods

The type specimen of *Tasmaniosaurus triassicus* (UTGD 54655) was studied at first-hand in the geological collection of the School of Earth Sciences of the University of Tasmania, Hobart (Tasmania, Australia), with the permission of the curator (see Acknowledgements).

All specimens that are used here for comparative purposes (indicated by the citation of their taxonomic name and respective collection accession numbers at relevant points in the manuscript) were studied at first-hand, with the explicit permission of appropriate curators and/or collection managers (see Acknowledgements), in recognized, scientifically accessible collections. Repository locations and abbreviations for all specimens discussed in the text and abbreviations listed in the Acknowledgements are as follows:

AM, Albany Museum, Grahamstown, South Africa; AMNH, American Museum of Natural History, New York, USA; BP, Evolutionary Studies Institute of the University of the Witswatersrand, Johannesburg, South Africa; BSPG, Bayerische Staatssammlung für Paläontologie und Geologie, Munich, Germany; GHG, Geological Survey, Pretoria, South Africa; IVPP, Institute of Vertebrate Paleontology and Paleoanthropology, Beijing, China; MB, Museum für Naturkunde, Berlin, Germany; MCNAM, Museo de Ciencias Naturales y Antropológicas de Mendoza (J. C. Moyano), Mendoza, Argentina; MCZ, Museum of Comparative Zoology, Harvard, USA; NHMUK, The Natural History Museum, London, UK; NM, National Museum, Bloemfontein, South Africa; NMK, Naturkundemuseum im Ottoneum Kassel, Kassel, Germany; PIMUZ, Paläontologisches Institut und Museum der Universität Zürich, Zurich, Switzerland; PIN, Paleontological Institute of the Russian Academy of Sciences, Moscow, Russia; PULR, Paleontología, Universidad Nacional de La Rioja, La Rioja, Argentina; PVL, Paleontología de Vertebrados, Instituto “Miguel Lillo”, San Miguel de Tucumán, Argentina; PVSJ, División de Paleontología de Vertebrados del Museo de Ciencias Naturales y Universidad Nacional de San Juan, San Juan, Argentina; QM, Queensland Museum, Brisbane, Queensland, Australia; RC, Rubidge Collection, Wellwood, Graaff-Reinet, South Africa; SAM-PK, Iziko South African Museum, Cape Town, South Africa; TM, Ditsong National Museum of Natural History (formerly Transvaal Museum), Pretoria, South Africa; UA, University of Antananarivo, Antananarivo, Madagascar; UMZC, University Museum of Zoology, Cambridge, UK; USNM, National Museum of Natural History, Smithsonian Institution, Washington DC, USA; UTGD, School of Earth Sciences, University of Tasmania, Hobart, Australia; WMSN, Westfälisches Museum für Naturkunde, Münster, Germany.

No specimens were purchased, donated or loaned for the purpose of this study. No permits were required for the described study, which complied with all relevant regulations.

Comparisons with the other known Australian proterosuchian *Kalisuchus rewanensis* are limited only to the holotype maxilla (QM F8998) because the assignment of the referred bones to the same species is questionable (e.g. Thulborn reported that the cranial bones belonged to different individuals because they are of disparate sizes and were collected on different occasions; [37]: 332) and will be discussed in detail elsewhere. However, it should be mentioned here that the holotype maxilla of *Kalisuchus rewanensis* was misinterpreted by Thulborn [37] as a right maxilla, but actually belongs to the left side of the skull as indicated by the presence of a palatal process of the maxilla on the medial surface of the bone (and not on the lateral surface as implied by the original interpretation).

Measurements were made with a digital caliper set with a maximum deviation of 0.02 mm but measurements were rounded to the nearest 0.1 millimetre. Values given between brackets in Tables 2–10 indicate incomplete measurements (due to post-mortem damage), whereas those with an asterisk indicate uncertain values (due to post-mortem deformation) and the values are the maximum measurable except where detailed.

**Table 2:** Measurements of some cranial bones of *Tasmaniosaurus triassicus* (UTGD 54655) in millimetres.

**right premaxilla**	Length	(35.1)
	Length of premaxillary body	(19.0)
	Height of premaxillary body	8.6
	Apicobasal height of largest crown	5.2
	Mesiodistal length at base oflargest crown	2.3
	Length of posterior process	(16.8)
**left maxilla**	Maximum length	71.0
	Length of horizontal ramus	50.2
	Height of ascending process	(12.6)
	Apicobasal height of largest crown	5.1
	Mesiodistal length of largestcrown at base	2.7
**?right maxilla**	Maximum length	(35.1)
	Apicobasal height of largest crown	5.3
	Mesiodistal length of largestcrown at base	2.2
**right lacrimal**	Length	(31.7)
	Height	(28.9)
**right pterygoid**	Length	(19.7)
	Width	(4.2)

**Table 3:** Measurements of skull roof bones of *Tasmaniosaurus triassicus* (UTGD 54655) in millimetres.

**skull roof**	Fronto-parietal length	74.6
	Fronto-parietal length without posterolateral process of parietal	53.5
	Width of right parietal including posterolateral process	23.2
	Width of frontals at level of orbits	20.2
	Length of olfactory bulbs (interpretation A)	(11.6)
	Length of olfactory bulbs (interpretation B)	(13.3)
	Width across olfactory bulbs (interpretation A)	9.2
	Width across olfactory bulbs (interpretation B)	18.5
	Length of olfactory tract	14.5
	Minimum width of olfactory tract	3.3
	Length of cerebral hemispheres	17.3
	Width along cerebral hemispheres	12.4

Interpretation A corresponds to the hypothesis of small olfactory bulbs and interpretation B correspond to the hypothesis of large olfactory bulbs (see Discussion).

**Table 4:** Measurements of lower jaw bones of *Tasmaniosaurus triassicus* (UTGD 54655) in millimetres.

**left dentary (anterior end)**	Length	(15.4)
	Height	10.1
	Length of largest ventral foramen	1.7
	Height of largest crown	(4.7)
	Length of largest crown at base	2.8
**left dentary (main portion)**	Length	(75.1)
**right dentary**	Length	(101.2)
	Height	18.1
**right splenial**	Length	(83.2)
	Height	14.4

**Table 5:** Measurements of presacral vertebrae of *Tasmaniosaurus triassicus* (UTGD 54655) in millimetres.

**cervico-dorsal**	Height	(38.6)
	Height of posterior centrum margin	10.8
	Width of centrum	11.9
	Width of neural canal	7.5
	Height of neural canal	5.3
	Length along zygapophyses	–
	Width along postzygapophyses	10.5
	Length of diapophysis	(8.2)
	Height of neural spine	(12.4)
	Width of neural spine at distal tip	(4.9)
	Minimum width of neural spine	1.5
**anterior/middle** **dorsal**	Height	47.9
	Length of centrum	19.8
	Height of anterior centrum margin	15.4
	Height of posterior centrum margin	17.0
	Length along zygapophyses	28.4
	Height of neural spine	25.5
	Length of neural spine at base	17.2
	Maximum length of neural spinedistal end	21.0

The length along the zygapophyses is the maximum anteroposterior length between the anterior tips of the prezygapophyses and the posterior tips of the postzygapophyses.

**Table 6:** Measurements of the caudal vertebrae of *Tasmaniosaurus triassicus* (UTGD 54655) in millimetres.

sequence of four middle caudal vertebrae	A	B	C	D			
Length of centrum	(11.7)	15.9	15.8	16.1			
Height of centrum	6.3	6.6	6.7	6.5			
Maximum height	8.4	9.3	10.6	10.2			
**sequence of seven middle-caudal vertebrae**	**A**	**B**	**C**	**D**	**E**	**F**	**G**
Length of centrum	(6.9)	17.5	18.0	17.3	15.2	16.7	18.3

**Table 7:** Measurements of the haemal arches of *Tasmaniosaurus triassicus* (UTGD 54655) in millimetres.

**lateral view**	Height	33.0
	Depth of proximal end	4.1
	Depth of distal plate	9.5
**transverse section**	Height	(18.0)
	Width of proximal end	5.6
	Height of haemal canal	7.4
	Width of haemal canal	2.9

The measurements correspond to the haemal arch exposed in lateral view and the other preserved in transverse section.

**Table 8:** Measurements of interclavicle of *Tasmaniosaurus triassicus* (UTGD 54655) in millimetres.

**interclavicle**	Length	91.1
	Width of anterior end	46.8
	Length of posterior process	67.8
	Minimum width of posterior process	5.8
	Width of expansion of posterior process	8.9

**Table 9:** Measurements of ?femur and tibiae of *Tasmaniosaurus triassicus* (UTGD 54655) in millimetres.

**?femur**	Length	(102.5)
	Minimum dorsoventral depth of shaft	(7.8)
**tibia A**	Length	91.1
	Dorsoventral depth of proximal end	34.8
	Minimum dorsoventral depth of shaft	13.8
	Dorsoventral depth of distal end mould	18.4
**tibia B**	Length	(70.5)
	Dorsoventral depth of proximal end	(42.5)
	Minimum dorsoventral depth of shaft	10.2

**Table 10:** Measurements of pedal bones of *Tasmaniosaurus triassicus* (UTGD 54655) in millimetres.

**?metatarsal II**	Length	37.4
	Width of proximal end	(8.3)
	Width of distal end	9.7
**metatarsal V**	Length	21.7
	Width of proximal end	18.4
	Width of distal end	6.5
**proximal phalanx**	Length	(38.6)
	Length of distal trochlea	10.5
	Height of distal trochlea	12.2
	Length of collateral pit	4.8
	Height of collateral pit	4.5
**distal phalanx**	Length	11.2
	Width of proximal end	6.7
	Width of distal end	5.7
**ungual**	Length	12.4
	Height	(4.5)

## Results

### Systematic Palaeontology

Diapsida Osborn, 1903 [38] sensu Laurin 1991 [39].

Sauria Gauthier, 1984 [40] sensu Gauthier et al. 1988 [41].

Archosauromorpha Huene, 1946 [42] sensu Dilkes 1998 [1].

*Tasmaniosaurus triassicus* Camp & Banks, 1978 [19].

Figures 2–4, 5A, 6–17, 18A.

**Figure 2:**
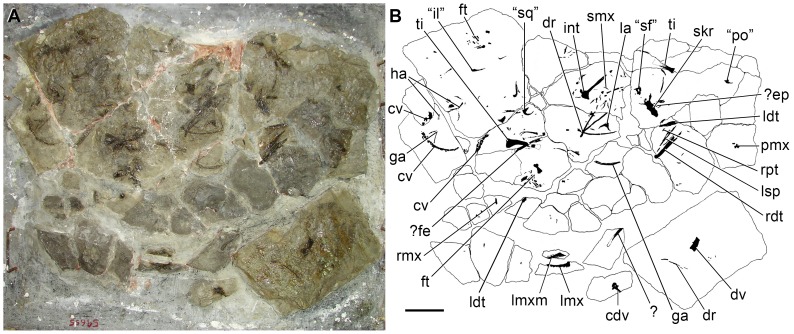
Slab including the artificially assembled blocks that compose the type specimen (UTGD 54655) of *Tasmaniosaurus triassicus*. **A**, photograph; **B**, line drawing. Abbreviations: “il”, ilium of Camp & Banks; “po”, postorbital of Camp & Banks; “sf”, supratemporal fenestra of Thulborn; “sq”, squamosal of Camp & Banks; ?, indeterminate bone; ?ep, epipterygoid; ?fe, probable femur; cdv, cervico-dorsal vertebra; cv, caudal vertebrae; dr, dorsal rib; dv, dorsal vertebra; int, interclavicle; ft, foot; ga, gastralia; ha, haemal arch; la, right lacrimal; ldt, left dentary; lmx, probable left maxilla; lmxm, probable left maxilla natural mould; lsp, left splenial; pmx, right premaxilla; rdt, right dentary; rmx, probable right maxilla; rpt, right pterygoid; ti, tibia; skr, skull roof; smx, small ?archosauriform maxilla. Scale bar equals 10 cm. Drawing of Fig. 2B modified from [19].

**Figure 3:**
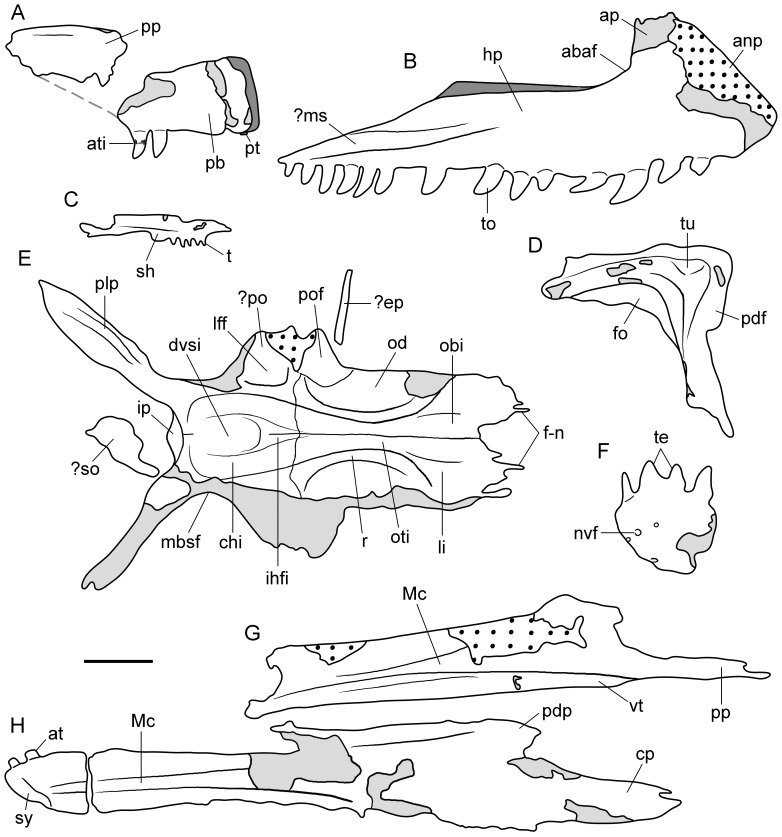
Line drawings of selected cranial bones of type specimen (UTGD 54655) of *Tasmaniosaurus triassicus*. **A**, right premaxilla in lateral view; **B**, left maxilla in probable medial view; **C**, partial right pterygoid and **D**, right lacrimal in medial views; **E**, skull roof elements in ventral view and possible supraoccipital and epipterygoid; **F**, anterior end of left dentary and **G**, left splenial in lateral views; and **H**, right dentary in medial view. Dotted areas are bone impressions, light grey areas are damaged bone, and dark grey areas are reconstructed bone. The light grey line in (A) is the reconstructed ventral mrgin of the posterior process of the premaxilla inferred from the slope of the anterodorsal margin of the left maxilla. Abbreviations: ?ep, possible epipterygoid; ?ms, possible medial shelf; ?po, probable postorbital; ?so, possible supraoccipital; abaf, anterior border of the antorbital fenestra; anp, anterior process; ap, ascending process; at, anterior tooth; ati, ankylothecodont tooth implantation; chi, cerebral hemisphere impression; cp, central posterior process of dentary; dvsi, dural venous sinus impression; f-n, fronto-nasal suture; hp, horizontal process; fo, fossa; ihfi, interhemispheral fissure impression; ip, interparietal; lff, laterosphenoid facet; li, lateral impression; mbsf, medial border of the supratemporal fenestra; Mc, Meckelian canal; nvf, neurovascular foramina; obi, olfactory bulb impression; od, orbital depression; oti, olfactory tract impression; pb, premaxillary body; pdf, posterodorsal flange; pdp, posterodorsal process of dentary; plp, posterolateral process of the parietal; pof, postfrontal; r, ridge; pp, posterior process; pt, premaxillary tooth; te, teeth; to, tooth; tu, tuberosity; sh, shelf; sy, symphysis; vt, ventral tuberosity. Scale bar equals 1 cm.

**Figure 4:**
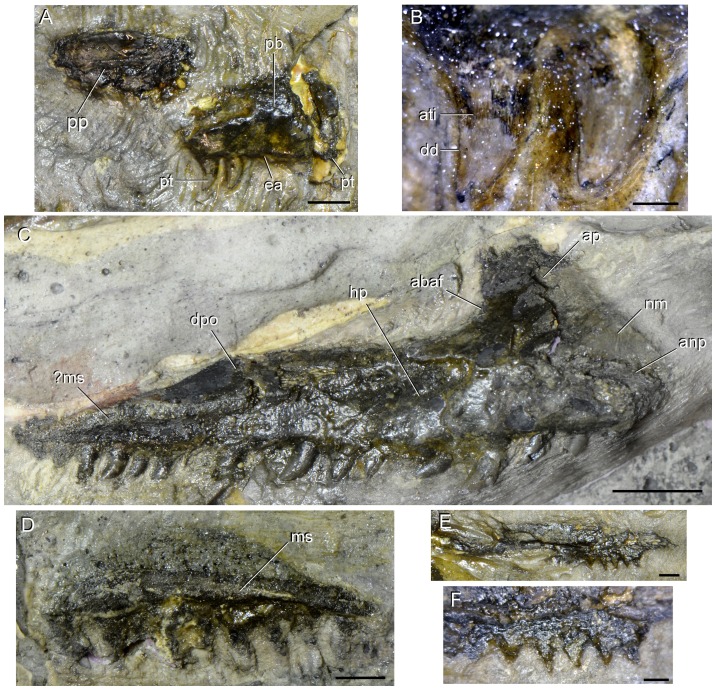
Tooth bearing-bones of type specimen (UTGD 54655) of *Tasmaniosaurus triassicus*. **A**, right premaxilla in lateral view and **B**, close up of the last two premaxillary teeth in labial view; **C**, left maxilla in probable and **D**, partial right maxilla in medial views; and **E**, partial right pterygoid and **F**, close up of T4 row of palatal teeth of pterygoid in medial views. Abbreviations: ?ms, possible medial shelf; abaf, anterior border of the antorbital fenestra; anp, anterior process; ap, ascending process; ati, ankylothecodont tooth implantation; dd, distal denticles; dpo, displaced portion; ea, empty alveolous; hp, horizontal process; ms, medial shelf; nm, natural mould; pb, premaxillary body; pp, posterior process; pt, premaxillary tooth; t, tooth. Scale bars equal 5 mm (A, D), 1 mm (B, F), 1 cm (C), and 2 mm (E).

**Figure 5:**
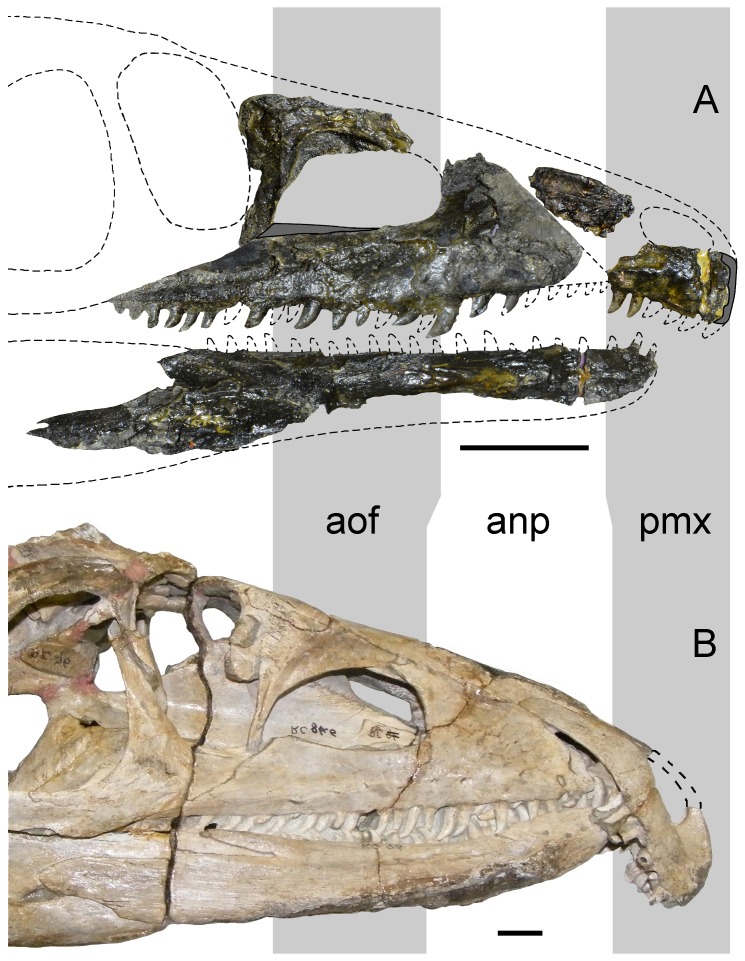
Reconstruction of the snout of *Tasmaniosaurus triassicus* (UTGD 54655) and comparison with the snout of *Proterosuchus fergusi* (RC 96). Snouts of **A**, *Tasmaniosaurus triassicus* and **B**, *Proterosuchus fergusi* (reversed) in lateral view. Note that all the bones of *Tasmaniosaurus triassicus* are shown in medial view, with exception of the premaxilla, which is shown in lateral view. Abbreviations: anp, anterior process of the maxilla length; aof, antorbital fenestra length; pmx, premaxilla length. Scale bars equal 1 cm (A) and 5 cm (B). Photograph of RC 96 courtersy of Fernando Abdala.

**Figure 6:**
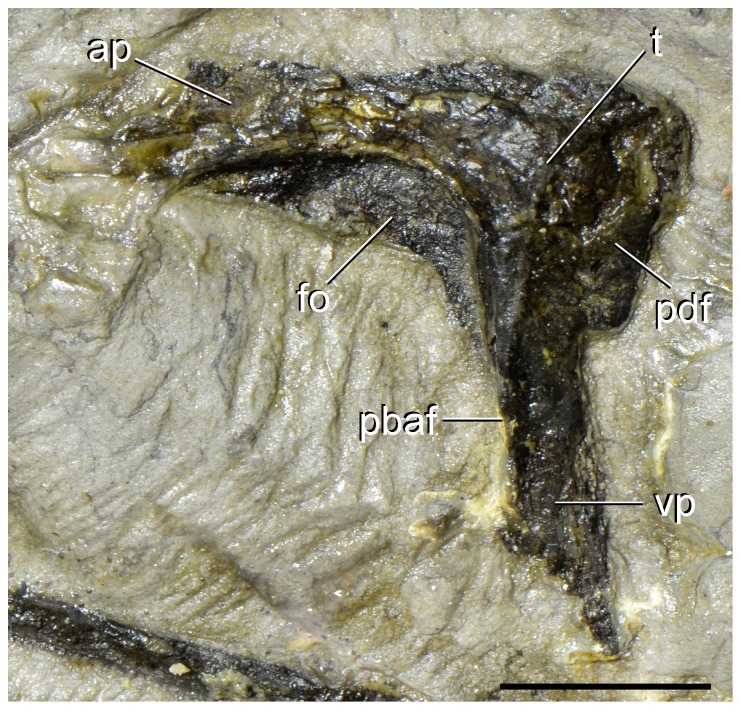
Right lacrimal of type specimen (UTGD 54655) of *Tasmaniosaurus triassicus* in medial view. Abbreviations: ap, anterior process; fo, fossa; pbaf, posterior border of the antorbital fenestra; pdf, posterodorsal flange; t, tuberosity; vp, ventral process. Scale bar equals 1 cm.

**Figure 7:**
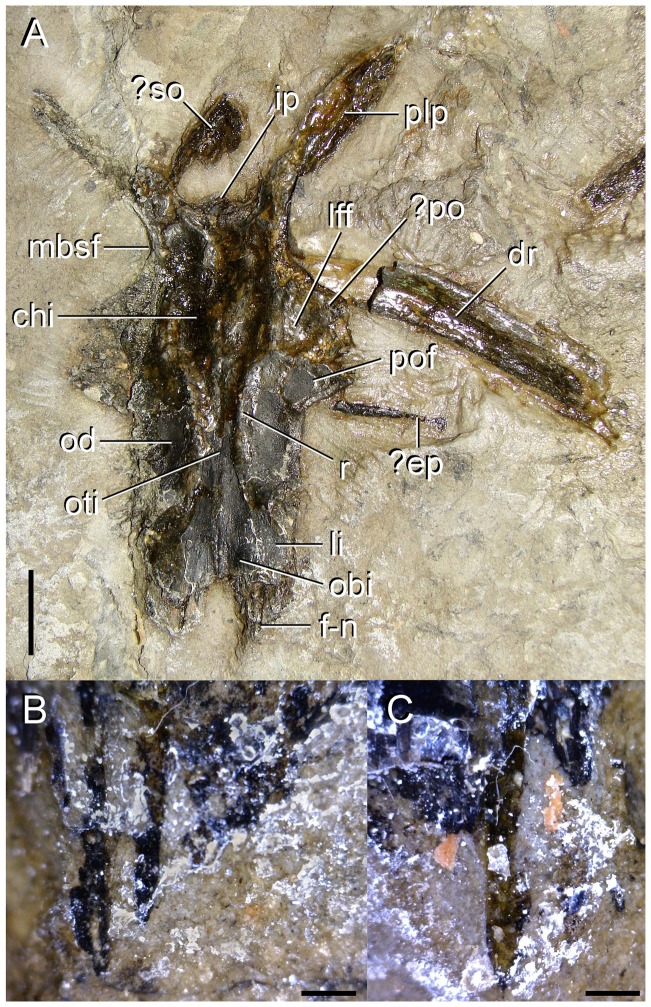
Skull roof and other bones of type specimen (UTGD 54655) of *Tasmaniosaurus triassicus*. **A**, **s**kull roof bones in ventral view and close-up views of the ventral surfaces of the anterior margins of the **B**, left and **C**, right frontals. Abbreviations: ?ep, possible epipterygoid; ?po, probable postorbital; ?so, possible supraoccipital; dr, dorsal rib; f-n, fronto-nasal suture; chi, cerebral hemisphere impression; ip, interparietal; lff, laterosphenoid facet; li, lateral impression; mbsf, medial border of the supratemporal fenestra; obi, olfactory bulb impression; od, orbital depression; oti, olfactory tract impression; plp, posterolateral process of the parietal; pof, postfrontal; r, ridge. Scale bars equal 1 cm (A) and 1 mm (B, C).

**Figure 8:**
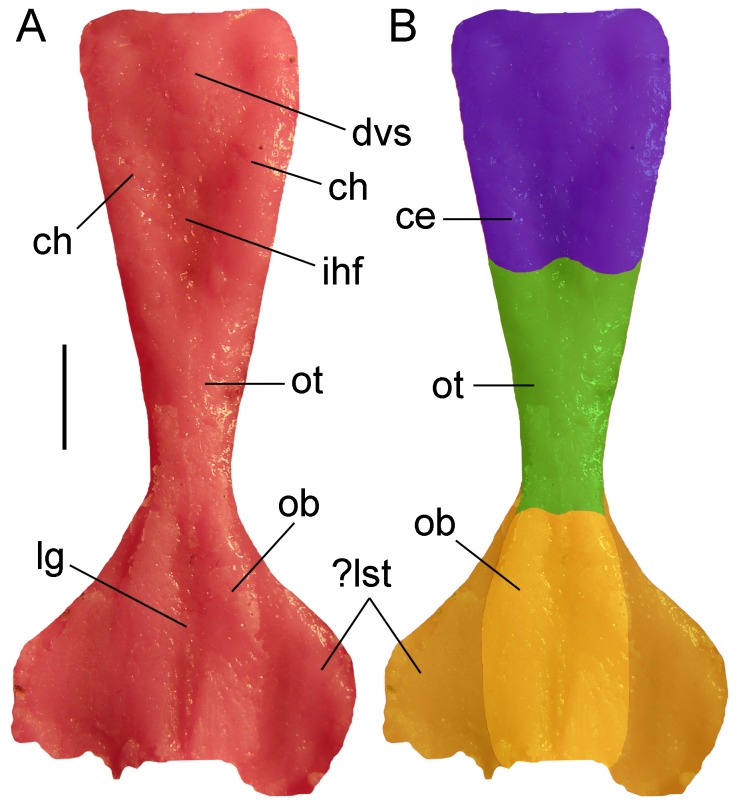
Latex cranial endocast of type specimen (UTGD 54655) of *Tasmaniosaurus triassicus*. **A**, latex endocast and **B**, interpretation of telencephalon areas in dorsal views. Cerebrum (blue), olfactory tract (green), olfactory bulbs (light yellow), and indeterminate soft tissue or lateral portion of the olfactory bulbs (dark yellow). Abbreviations: ?lst; lateral soft tissue or lateral portion of olfactory bulb; ce, cerebrum; ch, cerebral hemisphere; dvs, dural venous sinus; ihf, interhemispheral fissure; lg, longitudinal groove; ob, olfactory bulb; ot, olfactory tract. Scale bar equals 5 mm.

**Figure 9:**
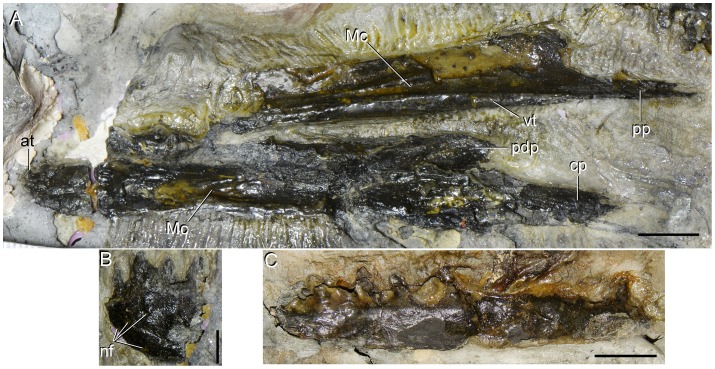
Mandibular bones of type specimen (UTGD 54655) of *Tasmaniosaurus triassicus*. **A**, right dentary and left splenial in medial and lateral views, respectively; **B**, anterior end of the left dentary in lateral view; and **C**, probable main portion of the left dentary in medial view. Abbreviations: cp, central posterior process of dentary; Mc, Meckelian canal; nf, neurovascular foramina; pdp, posterodorsal process of dentary; pp, posterior process of splenial; vt, ventral tuberosity. Scale bars equal 1 cm (A, C) and 5 mm (B).

**Figure 10:**
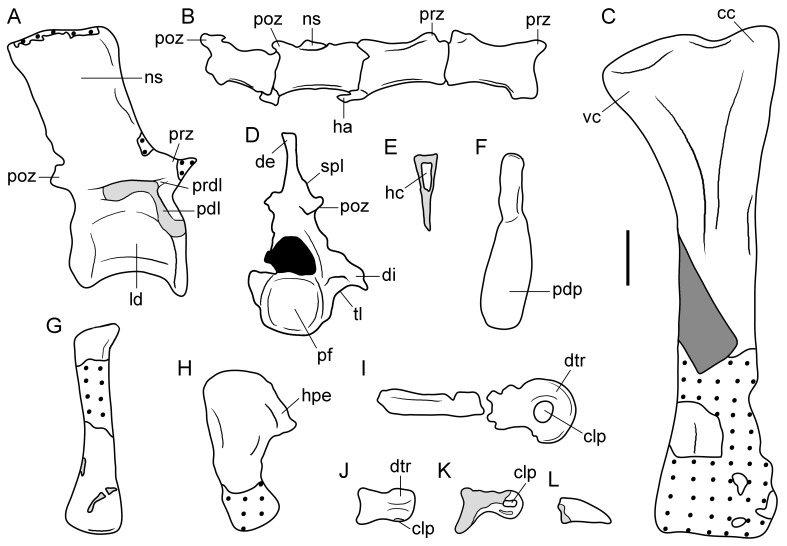
Line drawings of selected postcranial bones of type specimen (UTGD 54655) of *Tasmaniosaurus triassicus*. **A**, anterior or middle dorsal vertebra and **B**, middle caudal vertebrae in right lateral views; **C**, tibia in lateral or medial view; **D**, cervico-dorsal vertebra in posterior view; **E**, proximal half of haemal arch in crosss-section; **F**, anterior or middle haemal arch in right lateral view; **G**, probable metatarsal II and **H**, metatarsal V in dorsal or ventral views; **I**, proximal pedal phalanx in side view; **J**, pedal phalanx in ventral view; **K**, pedal phalanx in side view; and **L**, ungueal pedal phalanx in side view. Areas with dotted lines are bone impressions, light grey areas are damaged bone, and neural canal in black. Abbreviations: cc, cnemial crest; clp, collateral pit; de, possibly artificial distal transverse expansion; di, diapophysis; dtr, distal trochlea; ha, haemal arch; hc, haemal canal; hpe, hook-shaped proximal end; ld, lateral depression in the centrum; ns, neural spine; pdl, paradiapophyseal lamina; pdp, plate-like distal end; pf, posterior articular surface; poz, postzygapophysis; prz, prezygapophysis; prdl, prezygodiapophyseal lamina; spl, spinopostzygapophyseal lamina; tl, thick lamina; vc, ventral condyle. Scale bar equals 1 cm.

**Figure 11:**
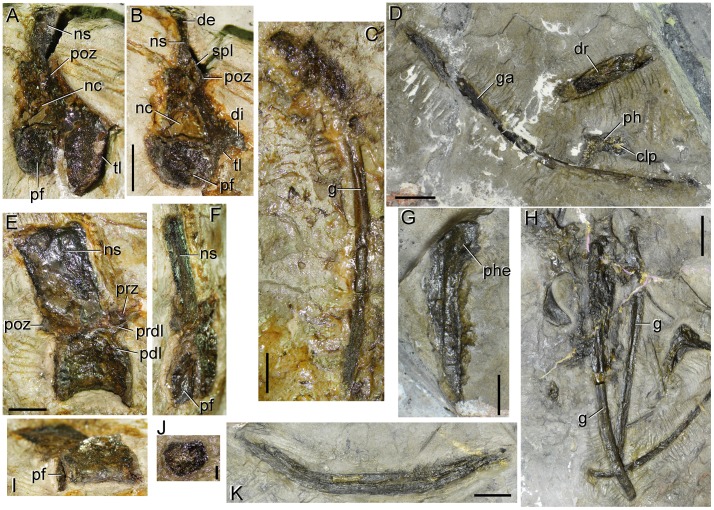
Postcranial presacral axial bones of type specimen (UTGD 54655) of *Tasmaniosaurus triassicus*. Cervico-dorsal vertebra in **A**, right posterolateral and **B**, posterior views. Anterior or middle dorsal vertebra in **E**, right lateral; **F**, mostly posterior; and **I**, mostly ventral views. **C**, **G**, **H**, dorsal ribs in posterior views. **D**, **K**, gastralia. **J**, possible intercentrum. Abbreviations: clp, collateral pit; de, possibly artificial distal transverse expansion; di, diapophysis; dr, dorsal rib; g, groove; ga, gastralium; nc, neural canal; ns, neural spine; pdl, paradiapophyseal lamina; pf, posterior articular surface; ph, pedal phalanx; phe, proximal dorsal rib head; poz, postzygapophysis; prdl, prezygodiapophyseal lamina; spl, spinopostzygapophyseal lamina; tl, thick lamina. Scale bars equal 1 cm (A–G, I, K), 2 cm (H) and 2 mm (J).

**Figure 12:**
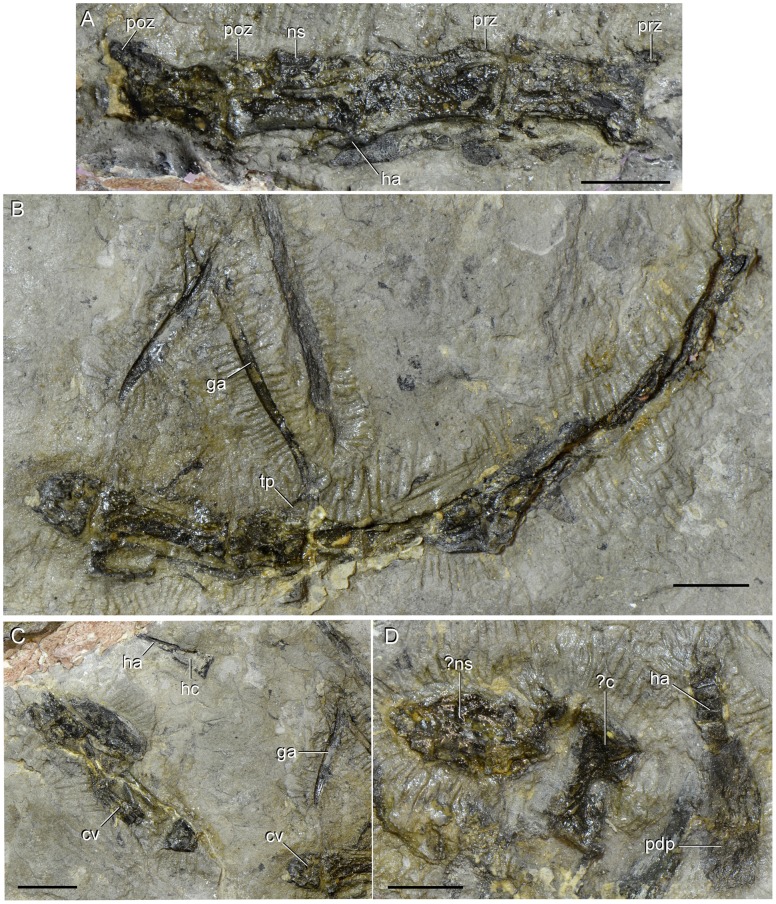
Caudal vertebrae and haemal arches of type specimen (UTGD 54655) of *Tasmaniosaurus triassicus*. **A**, middle caudal vertebrae in right lateral view; **B**, **C**, middle or distal caudal vertebrae in ventral and/or right posterolateral views; and **D**, probable anterior caudal vertebra in lateral view and anterior or middle haemal arch in right lateral view. Abbreviations: ?c, probable centrum; ?ns, probable neural spine; cv, caudal vertebra; ga, gastralium; ha, haemal arch; hc, haemal canal; ns, neural spine; pdp, plate-like distal end; poz, postzygapophysis; prz, prezygapophysis; tp, transverse process. Scale bars equal 1 cm.

**Figure 13:**
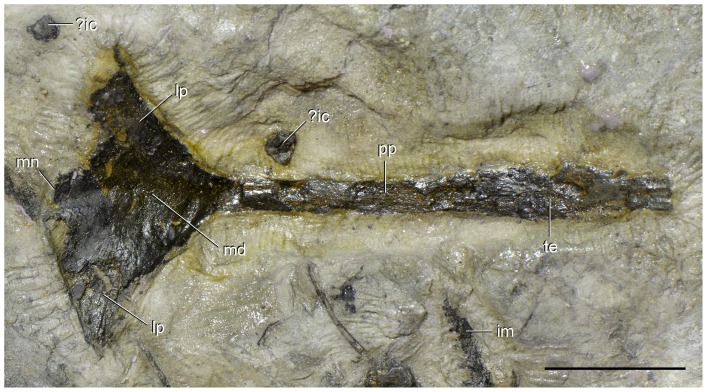
Interclavicle of type specimen (UTGD 54655) of *Tasmaniosaurus triassicus.* Interclavicle in dorsal view together with some possible intercentra and an isolated small maxilla. Abbreviations: ?ic, possible intercentrum; im, isolated small maxilla; lp, lateral process; md, median longitudinal depression; mn, median notch; pp, posterior process; te, transverse expansion. Scale bar equals 2 cm.

**Figure 14:**
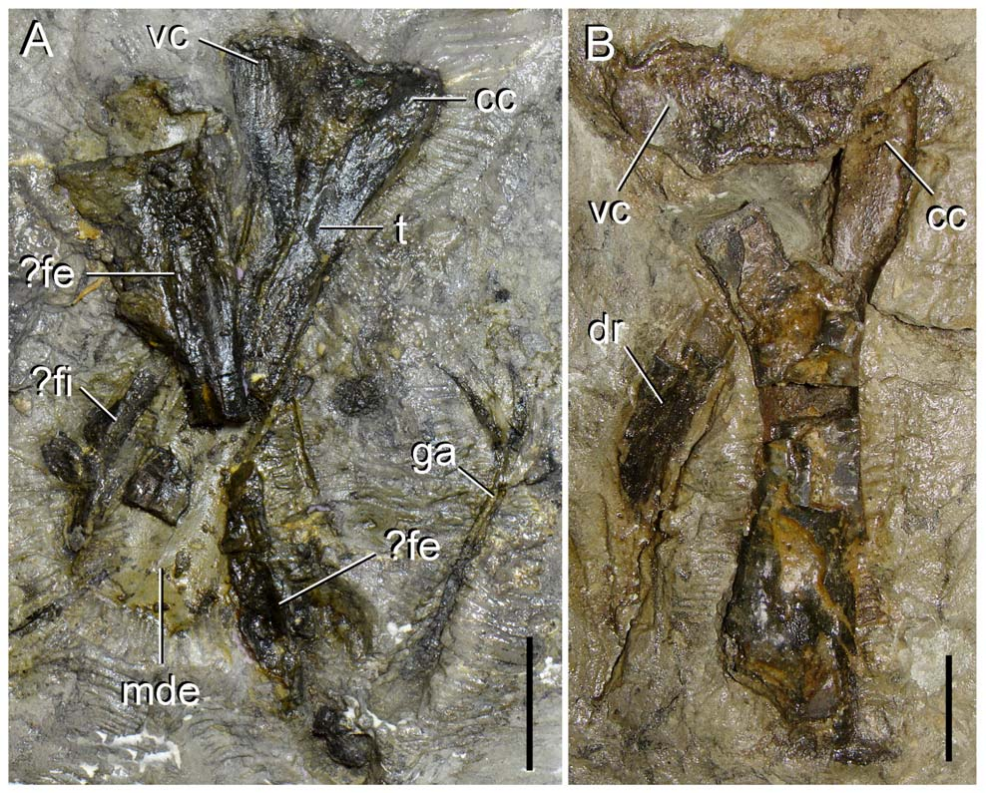
?Femur, tibiae and ?fibula of type specimen (UTGD 54655) of *Tasmaniosaurus triassicus*. **A**, ?femur and **A**, **B**, tibiae in lateral or medial views. Abbreviations: ?fe, femur; ?fi, fibula; cc, cnemial crest; dr, dorsal rib; ga, gastralia; mde, mould of distal end; t, tibia; vc, ventral condyle. Scale bars equal 2 cm (A) and 1 cm (B).

**Figure 15:**
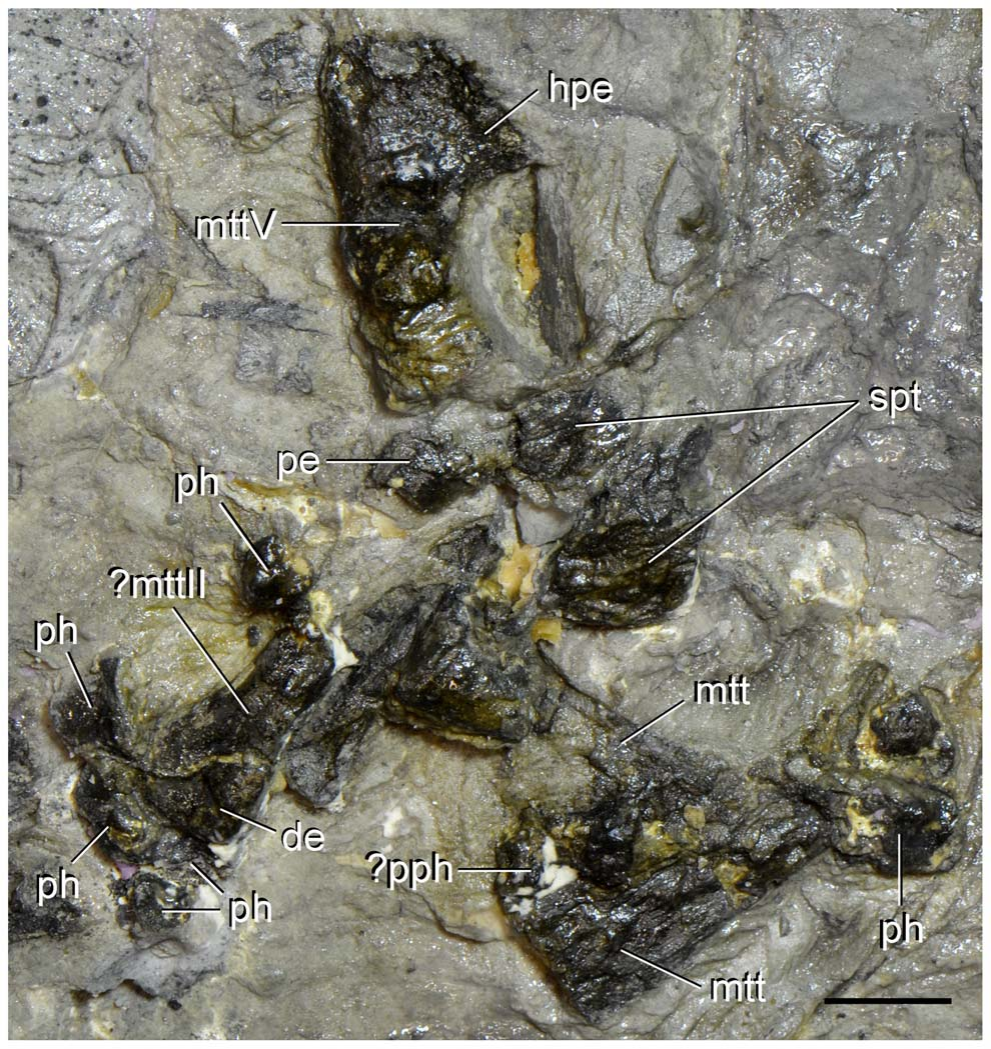
Pedal bones of type specimen (UTGD 54655) of *Tasmaniosaurus triassicus*. Abbreviations: ?mttII, probable metatarsal II; ?pph, probable proximal phalanx; de, distal end; hpe, hook-shaped proximal end; mtt, metatarsal; mttV, metatarsal V; pe, proximal end; ph, phalanx; spt, supposed proximal tarsals of Camp & Banks. Scale bar equals 1 cm.

**Figure 16:**
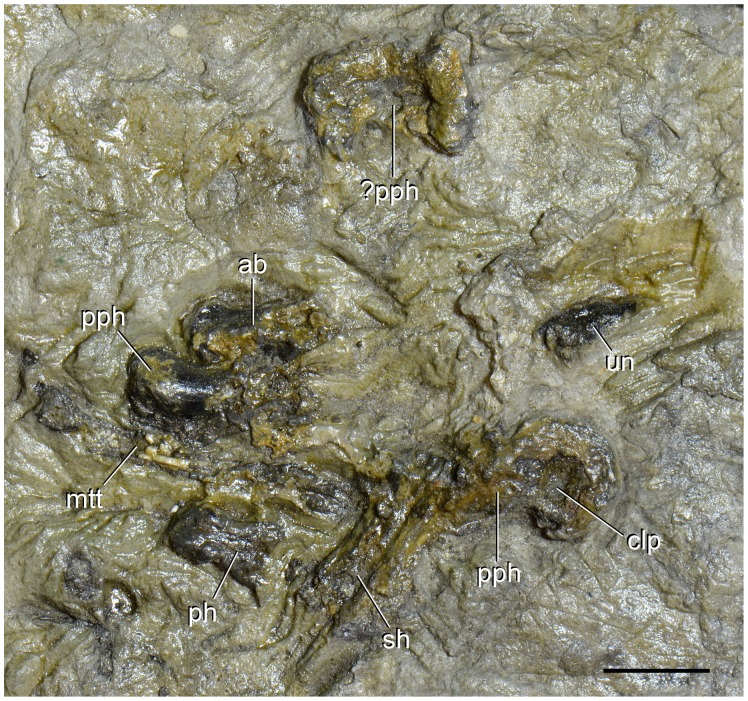
Pedal bones of type specimen (UTGD 54655) of *Tasmaniosaurus triassicus*. Abbreviations: ?pph, probable proximal phalanx; ab, autopodial bone; clp, collateral pit; mtt, metatarsal; pph, proximal phalanx; sh, shaft; un, ungueal. Scale bar equals 1 cm.

**Figure 17:**
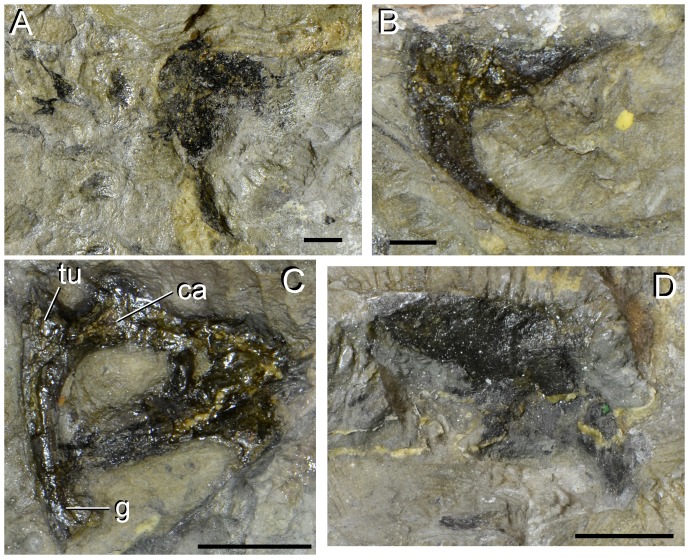
Problematic bones of type specimen (UTGD 54655) of *Tasmaniosaurus triassicus*. **A**, right postorbital of Camp & Banks; **B**, squamosal of Camp & Banks; **C**, “supratemporal fenestra” of Thulborn; and **D**, ilium of Camp & Banks. Abbreviations: ca, capitulum; g, groove; tu, tuberculum. Scale bars equal 5 mm (A, B) and 1 cm (C, D).

**Figure 18:**
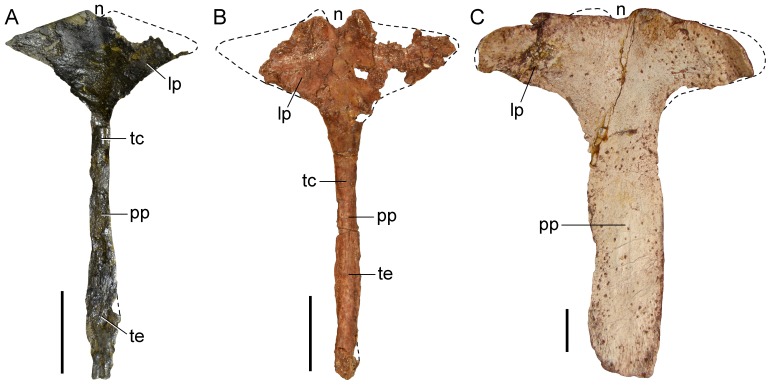
Comparisons of basal archosauromorph interclavicles in dorsal view. **A**, *Tasmaniosaurus triassicus* (UTGD 54655); **B**, *Prolacerta broomi* (BP/1/2675); and **C**, *Proterosuchus fergusi* (GHG 363). Abbreviations: lp, lateral process; n, median notch; pp, posterior process; tc, transverse compression; te, transverse expansion. Scale bars equal 1 cm.

“reptile related to *Chasmatosaurus*”; Banks 1962: unpaginated [43].

“*Chasmatosaurus* sp.”; Warren 1972∶281 [44].

#### Holotype

UTGD 54655, partial skeleton, mostly disarticulated, composed of the following elements: right premaxilla; left maxilla; probable right maxilla; right lacrimal; both frontals, postfrontals and parietals; interparietal; ?supraoccipital; right pterygoid; ?epipterygoid; both dentaries; left splenial; one cervico-dorsal and one anterior or middle dorsal vertebra; fourteen to sixteen caudal vertebrae; several ribs, gastralia and haemal arches; interclavicle; ?femur; both tibiae; and multiple metatarsals and pedal phalanges (Table 1). The different blocks that composes the holotype of *Tasmaniosaurus triassicus* are currently assembled within a plaster slab mount; however, the arrangement of individual blocks in this slab does not necessary reflect their actual original arrangement (Fig. 2). The artificial assembly of the different blocks in the slab mount is particularly evident in the case of the left maxilla. This bone is situated directly below its natural mould, and is thus artificially separated into two separate blocks in the slab mount but would actually have been a single block in situ. The vast majority of the bones possess the same kind of preservation, are of congruent size, there is no evidence of duplicate elements and all possess morphology congruent with that of a basal archosauromorph. Accordingly, these lines of evidence support the interpretation that almost all the elements included within UTGD 54655 belong to a single individual. However, the holotype of *Tasmaniosaurus triassicus* is mixed with, at least, an isolated maxilla of a considerably smaller animal [19]. Positive evidence could not be recognised for the presence of gut contents (contra Thulborn [20]; see below).

#### Referred specimens

Some bone fragments from other localities in Tasmania were previously referred to *Tasmaniosaurus triassicus* [22], [45] but could not be located in the collection of the UTGD in August 2012. Thulborn [20] considered these bones to be indeterminate.

#### Type horizon and locality

Crisp and Gunn’s Quarry at the head of Arthur Street (42°52′50.0″ S 147°18′10.6″ E ±100 metres) (Fig. 1), upper levels of the Poets Road Siltstone Member of the Knocklofty Formation (mostly correlated with the *Lystrosaurus* AZ of South Africa, Early Triassic, Induan–early Olenekian), Upper Parmeener Supergroup, Tasmania Basin, Hobart, Tasmania, Australia [19], [21] (see Geological and Palaeontological Setting).

#### Emended diagnosis

*Tasmaniosaurus triassicus* is a small-sized basal archosauromorph (skull length approximately 16 cm; based on a linear regression between skull and dentary length for the South African proterosuchid *Proterosuchus fergusi*, n = 11, R^2^ = 0.99) differentiated from other members of the clade by the following unique combination of characters (see below): premaxilla with posterodorsally oriented posterior process and ankylothecodont tooth implantation; maxilla with anteroposteriorly short anterior process; frontal with almost straight lateral margin; pterygoid with medial row of palatal teeth (modified from Camp & Banks [19]); dorsal vertebrae with paradiapophyseal and prezygodiapophyseal laminae and without distinct distal expansion of the neural spine; probable absence of osteoderms (Camp & Banks [19]); and interclavicle with a diamond-shaped anterior end and a gracile and slightly transversely expanded posterior process.

### Description

Several of the bones that comprise the holotype of *Tasmaniosaurus triassicus* suffered strong post-mortem compression and, in a number of cases, are currently covered by a dense layer of lacquer that prevents assessment of detailed anatomy and natural borders (e.g. right premaxilla). First hand study of the holotype of *Tasmaniosaurus triassicus* in August 2012 allowed a reassessment of several misinterpretations of bone identities made by Camp & Banks [19] and/or Thulborn [20] (Table 1), as well as the recognition of some structures and features that were overlooked by previous researchers. Moulds were made from several of the bones and allowed the recognition of further anatomical details, such as details of the ventral surface of the skull roof (i.e. cranial endocast).

### Cranium

#### Premaxilla

The preserved portion of the right premaxilla is exposed in lateral view and mostly congruent with the drawing of Thulborn ([20]: fig. 4a). That author corrected most of the original misinterpretations of Camp & Banks [19] (e.g. a reported premaxillary tooth count of 16) (Figs. 3A, 4A, B; Table 2). However, the anterior region of the premaxillary body is broken and anteromedially displaced from the rest of the bone. The drawing of Thulborn ([20]: fig. 4a) does not show this anteromedial displacement of the anterior end of the bone, and as a result, the premaxillary body appears artificially anteroposteriorly shorter in lateral view in his illustration than it would have been in life (Fig. 3A). Thus, the anteroposterior length of the premaxillary body of *Tasmaniosaurus triassicus* exceeded 2.21 times its dorsoventral height, resembling the condition present in *Protorosaurus speneri* (USNM 442453 cast of NMK S 180: ratio 2.59), *Prolacerta broomi* (BP/1/471: ratio 3.80), *Archosaurus rossicus* (PIN 1100/55: ratio 3.72), *Proterosuchus africanus* (RC 59: ratio 3.50; SAM-PK-11208: ratio 3.19; BP/1/3993: ratio 3.03; TM 201: ratio 3.03), *Sarmatosuchus otschevi* (PIN 2865/68: ratio 2.29) and *Euparkeria capensis* (UMZC T6921: ratio 2.61). By contrast, the premaxillary body of the erythrosuchids *Erythrosuchus africanus* (BP/1/5207 ratio 1.50; BP/1/4526: ratio 1.65), *Shansisuchus shansisuchus* ([46]: figs. 8, 9: ratio 1.07–1.33) and *Garjainia prima* (PIN 2394/5: ratio 1.82) is considerably anteroposteriorly shorter in comparison with its dorsoventral height.

The anterior margin of the premaxillary body is rounded and does not form a distinct acute angle with the alveolar margin (contra Thulborn [19]: fig. 4a). The condition of *Tasmaniosaurus triassicus* resembles that of *Prolacerta broomi* (BP/1/471), *Proterosuchus fergusi* (RC 59, BP/1/3993), *Erythrosuchus africanus* (BP/1/4526, 5207; NHMUK R3592), *Garjainia prima* (PIN 2394/5), *Euparkeria capensis* (SAM-PK-5867) and proterochampsids (e.g. *Chanaresuchus bonapartei*: MCZ 4037; PULR 07; *Gualosuchus reigi*: PULR 05). The lateral surface of the premaxillary body is convex, but due to the presence of a thick layer of lacquer it is impossible to assess more details of its anatomy (e.g. presence of neurovascular foramina). The postnarial process ( =  maxillary process or posterior process) of the premaxilla is partially preserved but is not in direct contact with the premaxillary body (Fig. 3A: pp; Fig. 4A: pp). However, it seems to be preserved in its original position with respect to the rest of the bone and its base was probably damaged during exposure of the fossil. This process is anteroposteriorly elongated and dorsoventrally tall, being subequal to the dorsoventral height of the preamxillary body, as also occurs in *Proterosuchus fergusi* (BP/1/3993; RC 59, 96; TM 201), “*Chasmatosaurus*” *yuani* (IVPP V90002, [47]), *Archosaurus rossicus* (PIN 1100/55), *Sarmatosuchus otschevi* (PIN 2865/68), *Erythrosuchus africanus* (BP/1/5207), *Garjainia prima* (PIN 2394/5) and *Euparkeria capensis* [48]. By contrast, the postnarial process is considerably lower than the height of the premaxillary body in *Protorosaurus speneri* [4], *Prolacerta broomi* (BP/1/471), *Fugusuchus hejiapanensis* ([49]: fig. 22), *Shansisuchus shansisuchus* (IVPP V2505, [46]) and proterochampsids (e.g. *Chanaresuchus bonapartei*: MCZ 4037; PULR 07; *Gualosuchus reigi*: PULR 05).

The preserved portion of the premaxilla and the slope of the anterodorsal margin of the maxilla indicate that if the long axis of the main body of the premaxilla is placed in a horizontal orientation, the postnarial process of the premaxilla would be posterodorsally oriented relative to the main body, resembling the condition of *Prolacerta broomi* (BP/1/471, [50]), *Erythrosuchus africanus* (BP/1/4526, 5207) and *Garjainia prima* (PIN 2394/5). By contrast, the postnarial process of small-sized *Proterosuchus fergusi* specimens (RC 59) is orientated parallel to the alveolar margin, and would be directed posteriorly if the main body of the premaxilla is held horizontally. Finally, in medium to large-sized *Proterosuchus fergusi* specimens (BP/1/3993; SAM-PK-11208; TM 201), *Archosaurus rossicus* (PIN 1100/55), “*Chasmatosaurus*” *yuani* (IVPP V90002, V4067) and *Sarmatosuchus otschevi* (PIN 2865/68) the postnarial process is directed posteroventrally if the main body of the premaxilla is held horizontally. The orientation of the postnarial process and the slope of the anterodorsal margin of the maxilla seem to indicate that the premaxilla of *Tasmaniosaurus triassicus* did not possess the extreme downturning observed in the above mentioned species (Fig. 5). Conversely, those features suggest that the premaxilla of *Tasmaniosaurus triassicus* would have been only slightly downturned, probably resembling the condition of *Prolacerta broomi* [50], *Garjainia prima* (PIN 2394/5) and *Erythrosuchus africanus* (BP/1/5207). The prenarial process ( =  nasal process, ascending process) of the premaxilla is completely missing and the ventral border of the external naris could not be distinguished. The palatal process of the premaxilla is either not preserved or not exposed.

The right premaxilla preserves three teeth in situ, one situated at the anterior end (in the anteromedially displaced anterior portion of the bone) (Fig. 3A: pt; Fig. 4A) and two positioned at the mid-length of the premaxillary body, at the posterior end of the alveolar margin. The most anterior tooth only preserves the base of its crown, whereas the two more posterior teeth possess completely preserved crowns (Fig. 4B). The anteromedially displaced anterior portion of the premaxillary body bears the partial crown and has room for another tooth position. In the main fragment of the premaxillary body, the probable presence of four or five tooth positions is estimated. Accordingly, the premaxilla of *Tasmaniosaurus triassicus* may have possessed a total of six or seven alveoli (Fig. 5A), resembling the condition of medium to large-sized *Proterosuchus fergusi* specimens (BP/1/3993; SAM-PK-K140; TM 201), *Sarmatosuchus otschevi* [51] and *Chanaresuchus bonapartei* [52]. By contrast, in small *Proterosuchus fergusi* specimens (RC 59), *Prolacerta broomi* [50] *Shansisuchus shansisuchus* [46], *Garjainia prima* (PIN 2394/5) and *Erythrosuchus africanus* [53] the premaxilla possesses five tooth positions.

In the most posteriorly preserved tooth of UTGD 54655, the crown is fused to the alveolar margin of the premaxilla via thin bony ridges (Fig. 3A:ati; Fig. 4B: ati), indicating the presence of an ankylothecodont tooth implantation, as also occurs in *Teraterpeton hrynewichorum* [54], *Prolacerta broomi* [50], *Proterosuchus fergusi* (BSPG 1934-VIII-514; RC 59; SAM-PK-11208; TM 201), and some teeth of *Azendohsaurus madagaskariensis* (UA 8-7-98-284) and *Garjainia triplicostata* (PIN 951/63). This crown also possesses denticles on at least its distal margin (Fig. 4B: dd), but evidence for denticles on the mesial margin could not be recognised. However, the mesial denticles of the premaxillary teeth of several basal archosauriforms are very small and restricted to the apical half of the crown (e.g. *Sarmatosuchus otschevi*: PIN 2865/68) and, as a result, the presence or absence of mesial denticles cannot be confidently assessed in *Tasmaniosaurus triassicus* because of the presence of a thick layer of lacquer covering the tooth and a poor state of preservation. The denticles are subrectangular in labial view and perpendicular to the main axis of the crown (Fig. 4B), as usually occurs in carnivorous archosauriforms. By contrast, the teeth of non-archosauriform diapsids are completely devoid of mesial or distal denticles (e.g. *Youngina capensis*: GHG K106; *Protorosaurus speneri*: [4]; *Macrocnemus bassanii*: PIMUZ T4822; *Prolacerta broomi*: BP/1/471). Both complete crowns are labiolingually compressed and slightly distally curved (with a convex mesial margin and a concave distal margin of the crown in labial view) without evidence of enamel ornamentation or ridges on their labial surfaces.

#### Maxilla

Thulborn [20] reported the presence of both maxillae and the natural mould of the most complete of the maxillae in UTGD 54655. A tooth-bearing bone interpreted by Camp & Banks [19] as the posterior part of the right premaxilla and as a probable fragment of maxilla by Thulborn [20] is identified here as the horizontal process of a partial right maxilla exposed in medial view (Fig. 4D; Table 2), because it possesses a distinct shelf that increases slightly in dorsoventral height anteriorly and is situated immediately above the alveolar margin of the bone (Fig. 4D: ms), which is also present in the medial surface of the maxilla of other archosauromorphs (e.g. *Prolacerta broomi*: BP/1/2675; *Kalisuchus rewanensis*: QM F8998). As a result, the more complete maxilla (Figs. 3B, 4C) described by Camp & Banks [19] and Thulborn [20] as an element from the right side is reinterpreted here as a left maxilla in medial view. In agreement with this interpretation is the presence of a longitudinally orientated thick, rounded shelf (possibly homologous to the shelf observed in the right maxilla) above the alveolar margin of the bone on the horizontal process of the maxilla (Fig. 4C: ?ms). The fragmentary condition of the right maxilla means that it is not possible to confirm that both shelfs are present in the same position on the horizontal process and can be considered as homologous structures. The interpretation of the most complete maxilla as a left element exposed in medial view is mainly a result of the identification of the other element as a right maxilla exposed in medial view and the presence of the shelf on the horizontal process. Accordingly, this interpretation should be considered with caution due to the poor preservation of the surface of the bone.

The natural mould of the left maxilla is currently mounted directly above the actual bone, which is clearly an artefact of the artificial assembly of the blocks within the plaster slab. The left maxilla lacks part of its anterior process and the distal end of the ascending process, but the anterior process is preserved as a natural impression adjacent to the actual bone, and as a natural mould on the counterpart (Fig. 4C: nm; Table 2). Indeed, on the counterpart there are still thin layers of bone that represent parts of this process, showing that it represents a reliable mould. The ascending process and the dorsal border of the horizontal process of the left maxilla are artificially laterally displaced from the rest of the bone as result of a longitudinal breakage (Fig. 4C: dpo). The left maxilla is moderately bowed medially along its entire length, a condition that it is not observed in the right maxilla. Thus, the curvature of the left bone seems to be the result of post-mortem deformation. A bone identified by Thulborn [20] as a left maxilla is here reinterpreted as a probable partial left dentary (see below).

The description of the maxilla is mostly based on the fairly complete maxilla probably exposed in medial view and the right maxilla does not provide substantial additional information. It should be noted that the original drawing of Camp & Banks ([19]: fig. 5f) of the most complete maxilla perfectly matches the condition observed in the specimen (Fig. 4C), but the drawing of Thulborn ([20]: fig. 5a) is strongly different. It seems to result from Thulborn [20] overlooking the ascending process and the natural impression of the anterior process of the maxilla.

The anterior process of *Tasmaniosaurus triassicus* is sub-triangular in medial view (Fig. 3B: anp; Fig. 4C: anp). It decreases in height anteriorly more abruptly than in *Proterosuchus fergusi* (RC 59, 96; BP/1/3993, 4016; SAM-PK-591, 11208, K140, K10603; TM 201; BSPG 1934-VIII-514; GHG 231) and “*Chasmatosaurus*” *yuani* (IVPP V90002, V4067). This condition cannot be determined in *Kalisuchus rewanensis* because the dorsal margin of the process is missing (QM F8998). The palatal process and the anterior end of the alveolar margin of the maxilla are not preserved in *Tasmaniosaurus triassicus*. The ascending process is sub-triangular and dorsally oriented, with a gently dorsoventrally concave posterior margin that defines the anterior border of the antorbital fenestra (Fig. 3B: abaf; Fig. 4C: abaf). The antorbital fenestra is also present in basal archosauriforms, such as *Proterosuchus fergusi* (RC 59, 96; BP/1/3993, 4016; SAM-PK-591, 11208, K140, K10603; TM 201; BSPG 1934-VIII-514; GHG 231), “*Chasmatosaurus*” *yuani* (IVPP V90002, V4067), *Fugusuchus hejiapanensis* ([49]: fig. 22), *Kalisuchus rewanensis* (QM F8998), erythrosuchids [46], [53], *Euparkeria capensis* [48] and proterochampsids [52], and crown archosaurs [13], [41]. By contrast, non-archosauriform diapsids such as *Prolacerta broomi* [50], *Protorosaurus speneri* [4], rhynchosaurs [1], *Trilophosaurus buettneri* [55], *Trilophosaurus jacobsi* [56], *Macrocnemus bassanii* (PIMUZ T4822) and *Tanystropheus longobardicus* [57] lack an antorbital fenestra. Furthermore, the ascending process of *Tasmaniosaurus triassicus* is not a vertical, pillar-like structure, contrasting with *Shansisuchus shansisuchus* (IVPP V2505), *Erythrosuchus africanus* (BP/1/5207), *Garjainia prima* (PIN 2394/5) and *Chalishevia cothurnata* (PIN 4356/1). The presence of an antorbital fossa cannot be assessed because the left maxilla is interpreted as being exposed in medial view.

The dorsal portion of the maxilla adjacent to the ventral border of the antorbital fenestra is broken and displaced laterally with respect to the rest of the bone. As a result, the dorsal margin of the horizontal process, along the border of the antorbital fenestra, appears to decrease in height posteriorly in lateral view (Fig. 4C). However, when the displaced portion of bone is reconstructed in the same plane as the rest of the bone, the horizontal process would have increased slightly in dorsoventral height posterior to the base of the ascending process (Fig. 3B). This condition is also present in *Proterosuchus fergusi* (RC 96; BP/1/4016; SAM-PK-591, 11208, K10603; TM 201; BSPG 1934-VIII-514; GHG 231), “*Chasmatosaurus*” *yuani* (IVPP V90002, V4067), *Kalisuchus rewanensis* (QM F8998) and *Fugusuchus hejiapanensis* ([49]: fig. 22). However, *Tasmaniosaurus triassicus* differs from the above mentioned taxa in possessing a straight ventral border of the antorbital fenestra, rather than a concave ventral border. Further posteriorly, the dorsal margin of the horizontal process of *Tasmaniosaurus triassicus* possesses a clear inflexion point, beyond which the whole process rapidly decreases in height posteriorly. The same condition is widespread among basal archosauriforms (e.g. *Proterosuchus fergusi*: RC 96; BP/1/4016; SAM-PK-591, 11208, K10603; TM 201; BSPG 1934-VIII-514; GHG 231; “*Chasmatosaurus*” *yuani*: IVPP V4067; *Fugusuchus hejiapanensis*: [49], fig. 22; *Euparkeria capensis*: SAM-PK-5867), in which immediately posterior to this inflexion point the maxilla contacts the ventral process of the lacrimal and the anterior tip of the jugal. The position of the inflexion point cannot be determined in *Kalisuchus rewanensis* because the posterior half of the dorsal margin of the horizontal process is missing (QM F8998). Accordingly, it is likely that the inflexion of the horizontal process indicates the posterior border of the antorbital fenestra (Fig. 5A). If this is indeed the case, then the length of the antorbital fenestra of *Tasmaniosaurus triassicus* seems to have been similar to that of *Proterosuchus fergusi* (RC 96; BP/1/4016; SAM-PK-591, 11208, K10603; TM 201; BSPG 1934-VIII-514; GHG 231) (Fig. 5B).

The tapering posterior end of the horizontal process indicates an extensive diagonal contact with the anterior process of the jugal [19], as in *Protorosaurus speneri* [4], *Prolacerta broomi* [50], *Proterosuchus fergusi* (RC 96; BP/1/4016; SAM-PK-591, 11208, K10603; TM 201; BSPG 1934-VIII-514; GHG 231), “*Chasmatosaurus*” *yuani* (IVPP V4067), *Fugusuchus hejiapanensis* ([49]: fig. 22), *Garjainia prima* (PIN 2394/5) and *Euparkeria capensis* (SAM-PK-5867). However, it cannot be determined whether or not the jugal participated in the border of the antorbital fenestra because the facet for the reception of this bone is not preserved on the left maxilla (contra Camp & Banks [19]). The alveolar margin of the left maxilla is almost straight along its entire length. The medial surfaces of both maxillae are heavily covered by lacquer and it is not possible to provide further details of the anatomy.

In the preserved portion of the alveolar margin of the left maxilla are 14 in situ teeth, with erupted crowns visible in medial view (Figs. 3B, 4C). The complete margin can be estimated to have held a total of 21 tooth positions [19], and to this count should be added the tooth positions belonging to the damaged anterior end of the bone (Fig. 5A). Accordingly, the complete tooth count of the maxilla of *Tasmaniosaurus triassicus* would have exceeded 21, as also occurs in medium to large-sized specimens of *Proterosuchus fergusi* (BP/1/3993; BSPG 1934-VIII-514; GHG 231; RC 96; SAM-PK-11208, K140, K10603; tooth count 22 to 31), “*Chasmatosaurus*” *yuani* (tooth count estimated as approximately 29; IVPP V90002, V4067) and *Prolacerta broomi* ([50]; BP/1/471; tooth count 24–25). *Kalisuchus rewanensis* preserves 14 tooth positions in the maxilla, but the complete tooth number cannot be determined because the posterior end of the bone is missing (QM F8998).

In the partial right maxilla of *Tasmaniosaurus triassicusi* nine teeth are preserved in situ (Fig. 4D). The tooth crowns are labiolingually compressed and distally curved, resembling the condition of other archosauriforms [1], [41]. The lingual surfaces of the crowns lack enamel ornamentation. It was not possible to discern any denticle on the mesial and distal margins of the preserved maxillary crowns, but Camp & Banks ([19]: 151) described the presence of “highly dentate carinae” in the maxillary teeth, as occurs in the premaxillary teeth. Thus, the denticles of the maxillary teeth are probably not currently observable due to the thick layer of lacquer that covers the crowns. The bases of the crowns seem to be fused to the bone of the maxillary alveolar margin without distinction between the crowns and the bone, suggesting an ankylothecodont tooth implantation, as is also the case in the premaxillary teeth. It is not possible to observe the tiny bony ridges that usually ankylose the crown to the bone in this kind of tooth implantation [13]. An alternate tooth replacement seems to be present in the anterior half of the left alveolar margin (Fig. 4C), as usually occurs in other archosauriforms [53], [58], but no tooth replacement pattern is observed among the posterior maxillary teeth.

#### Lacrimal

Camp & Banks [19] originally interpreted this bone as a left quadratojugal. Subsequently, Thulborn [20] noted clear discrepancies between this bone and archosauromorph quadratojugals and interpreted it as a probable composite element formed by parts of two or more gastralia. However, the element is composed of a single bone (contra Thulborn [20]). This bone is alternatively identified here as a right lacrimal exposed in medial view (Figs. 3D, 5; Table 2). The overall shape of the lacrimal closely resembles that of small to medium-sized specimens of *Proterosuchus fergusi* (BP/1/4016; SAM-PK-11208, K10603), with an angle between the anterior and ventral processes slightly higher than 90° and a similar shape of the concavity formed by the posterodorsal border of the antorbital fenestra. Furthermore, the size of the bone with respect to the premaxilla, maxillae, skull roof and mandibular bones is completely congruent with this interpretation (Figs. 3, 5). In particular, the lacrimal of *Tasmaniosaurus triassicus* is interpreted to be exposed in medial view because of the presence of a deep, well-defined fossa along the posterodorsal border of the antorbital fenestra, which is principally developed along the proximal half of the anterior process (Fig. 3D: fo; Fig. 6: fo), closely resembling the condition observed on the medial surface of the lacrimal of *Proterosuchus fergusi* (BP/1/4016; SAM-PK-11208). By contrast, the depression on the lateral surface of the lacrimal of *Proterosuchus fergusi* covers a proportionally larger area of the bone (BSPG 1934-VIII-514; GHG 231; RC 96), contrasting with the surface exposed on the lacrimal of *Tasmaniosaurus triassicus*. The lacrimal lacks the distal end of the anterior process and most of the distal end of the ventral process. The ventral process has a damaged posterior margin ( =  orbital margin).

The lacrimal of *Tasmaniosaurus triassicus* is an inverted L-shaped bone, resembling the condition of *Proterosuchus fergusi* (BSPG 1934-VIII-514; SAM-PK-11208, K10603), *Fugusuchus hejiapanensis* ([49]: fig. 22), *Garjainia prima* (PIN 2394/5) and *Erythrosuchus africanus* (BP/1/5207). By contrast, in the lacrimal of *Euparkeria capensis* (SAM-PK-5867) the anterior and ventral processes merge more smoothly into one another, and in non-archosauriform archosauromorphs the lacrimal is a sub-triangular bone (e.g. *Protorosaurus speneri*: [4]; *Trilophosaurus buettneri*: [55]; *Youngina capensis*: [59]; *Prolacerta broomi*: BP/1/471). The preserved portions of the anterior and ventral processes of the lacrimal are subequal in length, but because of the damaged ends it is not possible to determinate if the anterior process was longer than the ventral one as in *Proterosuchus fergusi* (BSPG 1934-VIII-514; BP/1/4016; SAM-PK 11208) and *Euparkeria capensis* (SAM-PK-5867). The anterior process is straight, transversely thin and tapers slightly towards its distal end (Fig. 6: ap), resembling the condition of some specimens of *Proterosuchus fergusi* (e.g. BSPG 1934-VIII-514). The posterodorsal corner of the lacrimal is sub-quadrangular and possesses a transversely thin, posteriorly extended flange that probably contacted the ventral process of the prefrontal (Fig. 3D: pdf; Fig. 6: pdf). The poor preservation of the bone surface prevents the identification of an articular facet for the prefrontal in this area. A similar flange, which is variably developed, is also present in some specimens of *Proterosuchus fergusi* (RC 96; SAM-PK-11208). The main body of the lacrimal (i.e. the portion at which the anterior and ventral processes converge) possesses a centrally placed, medially inflated tuberosity immediately next to the margin of the medial depression (Fig. 3D: tu; Fig. 6: t). This tuberosity disappears at the base of the anterior process but is well developed ventrally on the ventral process, delimiting the posterior border of the medial depression. The tuberosity merges gradually ventrally with the rest of the bone and disappears close to the mid-length of the ventral process. The medial surface of the ventral process posterior to the tuberosity is almost planar. The ventral process (Fig. 6: vp) tapers gradually distally, and due to its damaged posterior and distal margins it cannot be assessed if it was posteriorly curved or distally expanded. The ventral process forms the posterior border of the antorbital fenestra and it is slightly anteriorly concave (Fig. 6: pbaf).

#### Frontal

A partial skull roof with several bones in natural articulation is well preserved in the holotype of *Tasmaniosaurus triassicus* (Figs. 3E, 7; Table 3). Camp & Banks [19] originally described this skull roof as composed of the paired frontals, parietals, and postfrontals and the interparietal. However, Thulborn [20] reinterpreted this skull roof to be composed of the nasals, frontals and postorbitals. One of the main reasons for the reinterpretation of Thulborn [20] was the recognition by that author of a supposed fragment of the posterior end of the skull roof placed 3 cm away from the largest skull roof portion (a figure of the the skull roof and the supposed fragment of upper temporal region together and at the same scale is provided by Thulborn [20]: fig. 3a). This smaller fragment supposedly included one or both parietals and a squamosal and postorbital completely enclosing a supratemporal fenestra. The main portion of skull roof as interpreted by Thulborn [20] would possess a highly unusual morphology, mainly regarding the presence of an olfactory tract on the ventral surface of the nasals and posterolaterally divergent postorbitals. Re-examination of the specimen during the present study completely agrees with the original interpretation of Camp & Banks [19]. No evidence could be recognized to support the suggestion of Thulborn [20] that the bones situated 3 cm away from the skull roof are a parietal, squamosal or postorbital defining a supratemporal fenestra. Indeed, Thulborn ([20]: fig. 6) interpreted the lateral border of the supposed supratemporal fenestra to be mostly formed by the postorbital. However, the converse pattern is observed in basal archosauromorphs, in which a tapering anterior process of the squamosal lies medial to the posterior process of the postorbital and forms most of the lateral border of the supratemporal fenestra (e.g. *Proterosuchus fergusi*: SAM-PK-K10603; *Euparkeria capensis*: SAM-PK-5867). Additionally, in basal archosauromorphs the posterolateral process of the parietal possesses a sharp dorsal edge (e.g. *Proterosuchus fergusi*: SAM-PK-K10603; *Euparkeria capensis*: SAM-PK-5867) and is not a rod-like structure as it is the case in the supposed parietal described by Thulborn ([20]: fig. 6). Alternatively, as suggested here, the rod-like bone identified as a parietal by Thulborn [20] may represent the proximal half of a rib superimposed on an indeterminate fragment of bone (see below).

Thulborn [20] further mentioned some other features in the main portion of skull roof of *Tasmaniosaurus triassicus* to support the interpretation of the bones as nasals and frontals. These features deserve the following comments. Thulborn [20] described the anterior margin of the supposed nasals as possessing a V-shaped notch to receive the posterior tips of the prenarial process of the premaxillae, as is the case in many other archosauriforms. However, the morphology of the anterior border of the preserved portion of skull roof is completely consistent with that of the frontals of *Proterosuchus fergusi*. In *Proterosuchus fergusi* the nasal-frontal suture is strongly interdigitated and the nasals project posteriorly in between the frontals along the median line of the skull roof (e.g. SAM-PK-K10603). Additionally, Thulborn [20] described the presence of a lateral “cornice” in front of the orbit formed by the frontal and nasal. This lateral projection of the skull roof anterodorsal to the orbit is present in basal archosauriforms, but is mainly formed by the prefrontal and not by the nasal and frontal, contrasting with the original interpretation of Thulborn [20]. Conversely, the morphology of the “cornice” in *Tasmaniosaurus triassicus* is completely consistent with the lateral projection of the skull roof that articulates with the postorbital in other basal archosauriforms (e.g. *Prolacerta broomi*: BP/1/471; *Proterosuchus fergusi*: SAM-PK-K10603, RC 96; “*Chasmatosaurus*” *yuani*: IVPP V4067; *Euparkeria capensis*: SAM-PK-5867; *Chanaresuchus bonapartei*: PULR 07).

In the partial skull roof, the pair of frontals is almost complete, but the anterior and lateral borders of the left element are damaged (Figs. 3E, 7). An extensive longitudinal suture separates the frontals from each other (Fig. 3E). The pair of frontals of *Tasmaniosaurus triassicus* are together approximately 1.8 times longer than wide (width taken at the posterior level of the frontal orbital margin), resembling the anteroposteriorly-elongated frontals of other basal archosauromorphs (e.g. *Prolacerta broomi*: BP/1/471; *Proterosuchus fergusi*: RC 59). The frontals possess finger-like, well anteriorly developed projections for a strongly interdigitate articulation with the nasals (Fig. 3E: f-n; Fig. 7A: f-n, B, C), resembling the condition of small specimens of *Proterosuchus fergusi* (RC 59; SAM-PK-K10603). In the right frontal three distinct anterior projections can be recognised (Fig. 6B) of which the median is the longest. Although in the left frontal only two of these projections are discernable they possess the same morphology. In the best-preserved skull roofs of *Proterosuchus fergusi* each frontal has four (RC 59; SAM-PK-K10603) or five (RC 96) anterior projections that articulate with the nasal. In *Proterosuchus fergusi* the most lateral projection is immediately medial to (RC 59, 96; left frontal of SAM-PK-K10603) or adjacent to the suture with the prefrontal (right frontal of SAM-PK-K10603) and the most medial projection forms together with its counterpart on the opposite frontal a single median projection (RC 59, 96; SAM-PK-K10603). Regardless of individual variation, there are always three projections in the central region of the frontal in *Proterosuchus fergusi*, of which the medial one is the longest. Accordingly, the three anterior projections preserved at mid-width on the frontals of *Tasmaniosaurus triassicus* are in agreement with the intraspecific variation observed within *Proterosuchus fergusi* (RC 96). The overall shape of the fronto-nasal suture (e.g. W-shaped) cannot be assessed in *Tasmaniosaurus triassicus* because the anterior margins of both frontals are damaged at the mid-line of the skull roof. The lateral margin of the frontal is almost straight, up to its contact with the postfrontal. The postfrontal is not extended substantially anteriorly and, as a result, the frontal would have participated broadly in the dorsal border of the orbit (Figs. 3E, 7A), resembling the condition of *Protorosaurus speneri* [4], *Prolacerta broomi* (BP/1/471), *Proterosuchus fergusi* (RC 59, 96; SAM-PK-K10603), a referred specimen of *Archosaurus rossicus* (PIN 1100/48), *Sarmatosuchus otschevi* [50], *Euparkeria capensis* (SAM-PK-5867) and proterochampsids (e.g. *Chanaresuchus bonapartei*: PULR 07). By contrast, in erythrosuchids the frontal is excluded from the external border of the orbit (e.g. some specimens of *Shansisuchus shansisuchus* [46]) or has a limited participation in it (e.g. *Erythrosuchus africanus*: [53]; *Garjainia prima*: PIN 2394/5).

The suture with the postfrontal is situated at the posterolateral corner of the frontal and curves laterally, showing a very similar shape to that figured by Camp & Banks ([19]: fig. 5h) (Fig. 3E). The contact between the frontal and the postorbital on the ventral surface of the skull roof is not easily discernable due to the poor preservation of the bones in this area. However, following the shape of the postfrontal and the position of the suture between the frontal and parietal, the postorbital should have contacted the frontal in ventral view (Fig. 3E: ?po; Fig. 7A: ?po), as was figured by Camp & Banks ([19]: fig. 5h) and as occurs in other basal archosauromorphs (e.g. South African proterosuchid NM QR 880, referred to *Proterosuchus fergusi* by Welman [58]; *Erythrosuchus africanus*: NM QR 1473). The suture between the frontal and parietal is faint, transversely oriented and slightly interdigitated (Camp & Banks [19]) (Fig. 3E).

The ventral surface of the anterior end of the frontals possesses a well-preserved and distinct impression of the telencephalon, including the olfactory bulbs and tract and cerebrum (Fig. 3E: chi, obi, oti; Fig. 7A: chi, obi, oti), which have provided a reliable cranial endocast (Fig. 8). The impression, formed by the olfactory bulbs and possibly other soft tissue, covers the entire width of the anterior end of the paired frontals (Fig. 3E: li, obi; Fig. 7A: li, obi; Fig. 8: ?lst, ob), resembling the condition observed in *Prolacerta broomi* (BP/1/2675), *Archosaurus rossicus* (PIN 1100/48), *Sarmatosuchus otschevi* (PIN 2865/68), and *Erythrosuchus africanus* (NM QR 1473). The olfactory bulbs are positioned anterior to the level of the orbital depression of the frontals (Fig. 3E: obi; Fig. 7A: obi; Fig. 8: ob), but the interpretation of the morphology of the bulbs is ambiguous and will be discussed below. The olfactory bulbs lead posteriorly into the olfactory tract (Fig. 3E: oti; Fig. 7A: oti; Fig. 8: ot), which is located between both orbital depressions. The impression of the olfactory tract is hourglass-shaped in ventral view, with its narrowest area situated immediately posterior to the impression of the olfactory bulbs. A thick, semilunate ridge delimits the olfactory tract laterally and separates it from the gently concave surface of the orbital depression (Fig. 3E: r; Fig. 7A: r). The olfactory tract impression opens posteriorly into that of the cerebrum, which is extended along both frontals and parietals (Fig. 3E: chi; Fig. 7A: chi; Fig. 8: ce, ch). A sub-circular and large fossa is present on each side of the impression of the cerebrum, and extends along the frontal and parietal (Fig. 3E: lff; Fig. 7A: lff). A very similar fossa is also observed on the skull roof of a South African proterosuchid that probably does not represent *Proterosuchus fergusi* (NM QR 880) as well as in *Erythrosuchus africanus* (NM QR 1473), and in *Garjainia prima* the fossa receives the laterosphenoid (PIN 2394/5). As a result, it is likely that this fossa also received the laterosphenoid in *Tasmaniosaurus triassicus*, but it is not possible to assess if the laterosphenoid was ossified or not. The laterosphenoid facet is well delimited medially and separated from the cerebrum impression by a subtriangular inflated surface that contacts anteriorly the border of the orbital depression. The orbital depressions are well extended on the ventral surface of the frontals and at the level of their transversely widest point are approximately 3.3 times wider than the olfactory tract, resembling the condition of the crown-archosaurs *Coelophysis bauri* (USNM 529382: ratio ca. 3.2) and *Stagonolepis olenkae* ([60]: fig. 5). By contrast, in *Prolacerta broomi* (BP/1/2675: ratio ca. 1.7) and a South African proterosuchid (NM QR 880) the orbital depressions are considerably less extended medially onto the ventral surface of the frontal.

#### Postfrontal

The postfrontal delimits the posterodorsal border of the orbit (Fig. 3E: pof; Fig. 7A: pof), but the bone is not as anteriorly extended onto the ventral surface of the skull roof as in *Prolacerta broomi* (BP/1/2675). By contrast, the development of the postfrontal of *Tasmaniosaurus triassicus* is very similar to that of a South African proterosuchid (NM QR 880) and *Archosaurus rossicus* (PIN 1100/48). The suture between the right postfrontal and the frontal is clear, but its suture with the postorbital is not discernable because the area is damaged. On the left side of the skull roof the sutural contacts of the postfrontal are not preserved. The ventral surface of the postfrontal is concave and contributes to the posterolateral end of the orbital depression. It cannot be confidently determined whether or not in *Tasmaniosaurus triassicus* the parietal was excluded from contact with the postfrontal, as is the case in a South African proterosuchid (NM QR 880), or if these bones contacted each other, as occurs in *Prolacerta broomi* (BP/1/2675) and *Erythrosuchus africanus* (NM QR 1473).

#### Parietal

The right parietal is almost complete, whereas the posterolateral process is severely damaged in the left parietal (Figs. 3E, 7A; Table 3). A median, longitudinal suture separates the parietals from each other at their anterior end, but it is not preserved posteriorly with the exception of the area adjacent to the contact with the interparietal (Fig. 3E). The presence or absence of a pineal foramen cannot be confidently assessed because of the poor preservation of the parietals along the median surface of the skull roof. The parietal possesses a subtriangular anterolateral projection that forms the anteromedial border of the supratemporal fenestra and should articulate with the ascending process of the postorbital, but no suture is discernable in this area (Fig. 3E: ?po; Fig. 7A: ?po).

The lateral margin of the parietal is widely concave, defining the medial border of an anteroposteriorly elongate supratemporal fenestra (Fig. 3E: mbsf; Fig. 7A: mbsf), resembling the condition of several basal archosauromorphs (e.g. *Protorosaurus speneri*: [4]; *Tanystropheus longobardicus*: [57]; *Mesosuchus broomi*: SAM-PK-6536; *Prolacerta broomi*: BP/1/2675; *Proterosuchus fergusi*: BP/1/3993; SAM-PK-K10603; *Archosaurus rossicus*: PIN 1100/48; *Euparkeria capensis*: SAM-PK-5867). By contrast, in *Erythrosuchus africanus* (NM QR 1473), *Garjainia prima* (PIN 2394/5) and *Shansisuchus shansisuchus* [46] the medial border of the supratemporal fenestra is considerably deeper and more strongly concave, resulting in an anteroposteriorly shorter opening. The base of the posterolateral process of the parietal diverges at an angle of approximately 20° to the sagittal midline of the skull roof (Fig. 3E: plp; Fig. 7A: plp), but gradually bows laterally along its anterior half. The posterior half of the process is straight and tapers distally. The facet for the reception of the opisthotic is not discernable.

The parietals have a deep and concave impression of the telencephalon region of the brain and the latex cranial endocast has provided a reliable dorsal surface of the cerebrum (Fig. 8). Sampson & Witmer [61] pointed out that it is a reasonable assumption that the brain did not fill completely the endocranial cavity [62]–[64], but in the case of the telencephalon the endocast mostly represents the contour of the brain [61]. The dorsal surface of the telencephalon possesses a median, oval inflated area that seems to correspond to the dorsal longitudinal dural venous sinus (Fig. 3E: dvsi; Fig. 8: dvs), resembling the condition observed in crown archosaurs (e.g. *Majungasuarus crenatissimus*: [61]). However, evidence could not be recognised for a dural peak covering the posterior end of the cerebrum in *Tasmaniosaurus triassicus*, which is interpreted to mark the position of the pineal gland, contrasting with the situation reported for several theropod dinosaurs (e.g. *Majungasuarus crenatissimus*, *Allosaurus fragilis*, *Tyrannosaurus rex*; [61], [64]). Two anteroposteriorly elongated cerebral hemispheres (Fig. 3E: chi; Fig. 7A: chi; Fig. 8: ch) separated from each other by a median, shallow interhemispheral fissure (Fig. 3E: ihfi; Fig. 8: ihf) can be clearly identified. The long axes of the hemispheres are mostly longitudinally oriented, but with a small medial component, and are directed anteriorly towards the opening of the olfactory tract.

#### Interparietal

The interparietal of *Tasmaniosaurus triassicus* is relatively large and firmly sutured to both parietals (Fig. 3E: ip; Fig. 7A: ip), contrasting with the non-archosauriform archosauromorphs *Prolacerta broomi*, *Trilophosaurus buettneri* and *Mesosuchus broomi*, in which the interparietal is absent [1], [50]. The interparietal is a semilunate bone in ventral view, as a result of a posteriorly concave suture with the parietals. The lateral tip of the interparietal contacts the base of the posterolateral process of the parietal, as also occurs in *Proterosuchus fergusi* (SAM-PK-K10603), *Archosaurus rossicus* (PIN 1100/48) and *Fugusuchus hejiapanensis* ([49]: fig. 22). By contrast, in *Erythrosuchus africanus* the interparietal is more reduced in extent transversely (NM QR 1473). The posterior margin of the interparietal possesses a robust, rounded posterior projection, resembling the condition observed in *Proterosuchus fergusi* (RC 96; SAM-PK-K10603), *Erythrosuchus africanus* (BP/1/5207) and *Euparkeria capensis* (SAM-PK-5867).

#### Possible supraoccipital

Camp & Banks [19] identified a partial, thin bone situated a few millimetres away from the interparietal as a supraoccipital (Fig. 3E: ?so; Fig. 7A: ?so). Subsequently, Thulborn [20] reinterpreted this bone as the probable end of the partial rib shaft that lies next to the right parietal (Fig. 6A: dr). However, the shape of the bone does not match that of a rib head (capitulum or tuberculum) because it is too wide and planar (Fig. 6A). The size (width of 10.7 mm) of the bone closely resembles that expected for a supraoccipital. Furthermore, the position of the bone is strongly suggestive of a supraoccipital detached from the skull roof during burial. Accordingly, the original interpretation of Camp & Banks [19] is cautiously followed here. The partial supraoccipital is not very informative and no further details can be provided.

#### Possible epipterygoid

Camp & Banks [19] identified a slit-like bone preserved next to the right postfrontal as a partial epipterygoid (Fig. 3E: ?ep; Fig. 7A: ?ep). Thulborn [20] questioned this assignment, stating that there is no evidence to support the proposed identification. This bone, with a maximum preserved length of 13.4 mm, is too thin to represent a partial cervical rib shaft and too straight and gracile to be a fragment of hyoid. As a result, a possible explanation is that it represents an anteriorly displaced epipterygoid lacking its ventral end (cf. Camp & Banks [19]). However, as pointed out by Thulborn [20], the evidence supporting this interpretation is weak.

#### Pterygoid

A thin bone bearing some small teeth is preserved next to the possible partial left dentary (the left dentary was identified as the left maxilla by Camp & Banks [19] and Thulborn [20]) (Figs. 3C, 4E, F). Camp & Banks [19] interpreted this tooth-bearing bone as a probable right ectopterygoid, or less likely a vomer or fragment of pterygoid, and Thulborn [20] considered it as an indeterminate element. Camp & Banks [19] described the presence of five tiny teeth along one of the edges of the bone, but Thulborn [20] considered these projections to be misinterpreted needle-marks produced during preparation of the specimen. First hand observation confirmed that the bone does in fact have palatal teeth based on the following lines of evidence: i) the teeth are regularly spaced; ii) the teeth possess almost exactly the same shape and size along the preserved series; iii) the teeth possess a mustard-like to black colour, suggesting an enamel covering, as in the premaxillary, maxillary and dentary tooth crowns; and iv) the teeth are continuous with the bone surface and they are not well-defined depressions in the matrix as would be expected for needle-marks (Fig. 4F). Accordingly, the evidence clearly supports the original interpretation of Camp & Banks [19] instead of the re-interpretation of Thulborn [20]. The preserved portion of the bone can be tracked along a long extension (cf. Camp & Banks [19]: fig. 5m) and seems to be interrupted by an overlying natural mould of a long, curved bone. Thus, the morphology of the bone does not match that of an archosauromorph ectopterygoid because it is too long to represent a medial ( =  pterygoid) process and clearly differs from the strongly posteriorly bowed lateral process of an ectopterygoid. Furthermore, the ectopterygoid of archosauromorphs does not bear teeth (e.g. *Mesosuchus browni*: SAM-PK-6536; *Proterosuchus fergusi*: RC 59; *Euparkeria capensis*: [48]). In addition, the morphology of the bone does not match with that of a vomer or palatine because the orientation of the palatal teeth would result in a bone that is too dorsoventrally deep. By contrast, the morphology of the preserved portion of the bone is almost identical to that of the pterygoid of *Proterosuchus fergusi* (RC 59) and *Prolacerta broomi* (BP/1/2675). The preserved portion of bone seems not to belong to the lateral process of the pterygoid because in this region the palatal teeth are arranged perpendicular to the main plane of the process ([58]; e.g. *Proterosuchus fergusi*: SAM-PK-11208). Instead, the palatal teeth are oriented parallel to the main plane of the bone, as occurs in the anterior process of the pterygoid of *Prolacerta broomi* (BP/1/2675) and *Proterosuchus fergusi* (RC 59). Furthermore, this bone is laminar and possesses an upraised shelf immediately next to the dentigerous margin (Fig. 3C: sh). This shelf is observed on the medial surface of the anterior process of the pterygoid of *Prolacerta broomi* (BP/1/2675) and, as a result, the palatal bone of *Tasmaniosaurus triassicus* likely represents the anterior process of a pterygoid exposed in medial view (Tables 1, 2). The curvature of the palatal teeth indicates that the element represents a right pterygoid if the interpretation that it is exposed in medial view is correct.

The dentigerous margin possesses a series of six or, more probably, seven compressed and blade-like palatal teeth, which are interpreted to belong to the T4 row of pterygoid teeth (sensu Welman [58]). The row of palatal teeth should have continued along the non-preserved dentigerous margins of the bone, as is the case in *Prolacerta broomi* (BP/1/2675) and *Proterosuchus fergusi* (RC 59). Each tooth has an apicobasal height of 0.7–0.8 mm and the best-preserved teeth are slightly distally curved, as is the case in the T4 teeth of *Prolacerta broomi* (BP/1/2675) and *Proterosuchus fergusi* (RC 59). No clear distinction is evident between the teeth and the tooth-bearing bone, implying that the teeth were probably ankylosed to the bone. However, due to the poor state of preservation of the element this interpretation should be considerate tentative. Similarly, due to preservation it could not be ascertained whether additional tooth rows are present on the rest of the anterior process of the pterygoid, such as the T3 row that lies immediately lateral to the T4 row in *Proterosuchus fergusi* [58].

The bone identified by Camp & Banks ([19]: fig. 5l) as a pterygoid is here interpreted as an indeterminate element, in agreement with Thulborn [20]. Camp & Banks [19] described the presence of at least four, sharply pointed small teeth along the medial and posterior borders of the supposed right pterygoid. However, these palatal teeth seem to be misidentified and instead represent irregular needle-marks produced during preparation. Although Thulborn [20] erroneously suggested the same interpretation for the “ectopterygoid” teeth identified by Camp & Banks [19] (see above), in the case of the supposed pterygoid teeth he did not raise a similar objection and merely stated that the teeth could not be identified, probably because they were concealed by lacquer. The rest of the bone is planar, long, and is, at least partially, still covered by matrix, with a maximum exposed linear dimension of 56.7 mm. Two needle-marks expose part of the covered surface of the bone and artificially appear like tiny, black palatal teeth. The identification of the bone remains elusive.

#### Dentary

Both dentaries, a fairly complete right dentary (Figs. 3H, 9A) and a partial left dentary (Fig. 9B, C), are preserved in the holotype of *Tasmaniosaurus triassicus* (contra Camp & Banks [19]) (Table 4). In contrast to Thulborn [20], the supposed right mandible of that author is interpreted here as a left splenial in lateral view (Figs. 3G, 9A), in agreement with Camp & Banks [19]. This left splenial is positioned close to the right dentary (see below). The bone described by Thulborn [20] as a left maxilla is instead re-interpreted here as a partial left dentary exposed in medial view (Fig. 9C) because it possess an alveolar margin very slightly longer than the other fairly complete maxilla (Tables 2, 4), and if it belongs to part of the right maxilla it would result in a maxilla considerably longer than the fairly complete counterpart. Moreover, there is no evidence of a tapering posterior end, as it should be expected in a maxilla, and the overall shape and size of this tooth-bearing bone matches very well with that of the right dentary. Because the right dentary is exposed in medial view, this bone is interpreted as a probable left dentary exposed in medial view. Both dentaries are preserved relatively close to each other in the same block. The partial bone identified by Camp & Banks [19] as a right premaxilla and by Thulborn [20] as the anterior end of the right dentary is alternatively reinterpreted here as the anterior end of the left dentary exposed in lateral view (Figs. 3F, 9B). This bone does not belong to a premaxilla (contra Camp & Banks [19]) because there is no evidence for the presence of an ascending process along the well-preserved border opposite to the alveolar margin and the margin that should have formed the narial border is continuously convex. Furthermore, the slightly convex surface of the bone does not match the more strongly convex lateral surface expected for a premaxilla. Alternatively, the overall morphology of this partial bone matches perfectly with that of the anterior end of a dentary [20]. The exposed surface of the bone possesses four large and oval foramina, in which three of them aligned parallel to the alveolar margin of the bone. A series of identical foramina in the same position are also present in the dentary of *Proterosuchus fergusi* (BSPG 1934-VIII-514; RC 59, 96; SAM-PK-K10603) and a dentary referred to *Archosaurus rossicus* (PIN 1100/48). Furthermore, there is no evidence of a symphyseal facet on this bone as should be expected on the medial surface of the dentary. Accordingly, this anterior end of dentary is interpreted as a left element exposed in lateral view (contra Thulborn [20]). Finally, the fairly complete dentary that Thulborn [20] identified as a left element exposed in lateral view is re-interpreted here as a right dentary exposed in medial view because of the presence of a long and extensive Meckelian canal along the surface of the bone (Fig. 3H: Mc; Fig. 9A: Mc). The bone that Thulborn ([20]: fig. 5) interpreted as a displaced left splenial seems to be a composite formed by part of the right dentary and possibly the poorly preserved anterior end of the left splenial or another fragment of bone (Figs. 3H, 9A). The description of the dentary is based on the right bone and anterior end of the left bone; the more complete portion of probable left dentary does not provide more information than the right dentary.

The dentary of *Tasmaniosaurus triassicus* is an anteroposteriorly-elongated bone, being approximately 11.1 times longer than the dorsoventral height of its anterior end (Figs. 3H, 9A). Thus, the dentary resembles that of *Protorosaurus speneri* [4], *Prolacerta broomi* (BP/1/2675), “*Chasmatosaurus*” *yuani* (IVPP V4067), and *Proterosuchus fergusi* (BP/1/4016: ratio 10.4; SAM-PK-K10603: ratio 11.6; RC 96: ratio 10.6; GHG 231: ratio 10.5) in its gracility. By contrast, the dentary is more robust in *Euparkeria capensis* (SAM-PK-5867: ratio 7.8) and *Erythrosuchus africanus* (BP/1/5207: ratio 5.0). The dentary of *Tasmaniosaurus triassicus* is mostly straight in medial view (Figs. 3H, 9A), resembling the condition of *Prolacerta broomi* (BP/1/471), *Erythrosuchus africanus* (BP/1/5207) and *Shansisuchus shansisuchus* [46]. By contrast, in *Proterosuchus fergusi* (BP/1/3993; BSPG 1934-VIII-514; RC 59; SAM-PK-11208; TM 201), “*Chasmatosaurus*” *yuani* (IVPP V90002, V4067), a dentary referred to *Archosaurus rossicus* (PIN 1100/78), *Sarmatosuchus otschevi* (PIN 2865/68), *Garjainia prima* (PIN 2394/5) and *Euparkeria capensis* (SAM-PK-5867) the dentaries are distinctly dorsally curved towards the anterior end. The ventral margin of the dentary is almost straight along most of its length and becomes slightly ventrally concave at its posterior end as result of the dorsoventral expansion of the bone. The alveolar margin is not well preserved in the fairly complete right dentary, but it seems to be straight or slightly concave. In both dentaries the anterior end of the alveolar margin curves gradually ventrally (Figs. 3F, H, 9A, B), resulting in a distinctly anterodorsally oriented first dentary tooth (Fig. 3H: at; Fig. 9A: at), resembling the condition observed in *Proterosuchus fergusi* (RC 96). The posterior end of the right dentary possesses two distinct processes, one dorsal (Fig. 3H: pdp; Fig. 9A: pdp) and one ventral (Fig. 3H: cp; Fig. 9A: cp). The ventral process is considered homologous to the process that forms the anterodorsal border of the external mandibular fenestra in *Proterosuchus fergusi* (RC 96) and *Erythrosuchus africanus* (BP/1/5207). In *Erythrosuchus africanus* the dentary possesses three posterior processes and the process that participates in the anterodorsal border of the fenestra corresponds to the central posterior process. As a result, following the morphology present in *Erythrosuchus africanus*, the two posterior processes present in *Proterosuchus africanus* and *Tasmaniosaurus triassicus* are termed posterodorsal and central posterior process, respectively. The posterodorsal process of *Tasmaniosaurus triassicus* is broken off distally and the central posterior process is well extended posteriorly, resembling the condition of *Proterosuchus fergusi* (RC 96; SAM-PK-K10603), but contrasting with the considerably shorter processes of *Erythrosuchus africanus* (BP/1/5207) and *Euparkeria capensis* (SAM-PK-5867). It cannot be assessed if the central posterior process participated in the anterior border of the external mandibular fenestra (if present), as is the case in *Proterosuchus fergusi* (RC 96; SAM-PK-K10603).

The length of the alveolar margin of the right dentary is roughly equal to that of the left maxilla. In basal archosauromorphs the dentary alveolar margin does not extend posteriorly beyond the posterior end of the maxillary alveolar margin (e.g. *Prolacerta broomi*: BP/1/471; *Proterosuchus fergusi*: RC 96; *Garjainia prima*: PIN 2394/5; *Euparkeria capensis*: [48]). Thus, if in *Tasmaniosaurus triassicus* the anterior tip of the dentary was situated level with the posterior margin of the alveolar margin of the premaxilla, as in *Proterosuchus fergusi* (RC 96) (Fig. 5B) and *Prolacerta broomi* (BP/1/471), the dentary alveolar margin would have ended posteriorly at the same level level as the maxillary alveolar margin, contrasting with the condition widespread among basal archosauromorphs in which the maxillary alveolar margin extends further posteriorly than the dentary alveolar margin. Accordingly, the relative lengths of the maxilla and dentary of *Tasmaniosaurus triassicus* suggest that the anterior tip of the lower jaw would have been situated slightly posterior to or at the same level as the anterior end of the premaxillary alveolar margin (Fig. 5A), as is the case in *Erythrosuchus africanus* (BP/1/5207), *Garjainia prima* (PIN 2394/5) and *Euparkeria capensis* (SAM-PK-5867), but differing from the condition in *Proterosuchus fergusi* (BP/1/3993, SAM-PK-11208, RC 96) and *Prolacerta broomi* (BP/1/471).

The lateral surface of the dentary is only known from the anterior end of the left bone. This surface possesses three large and oval foramina that are aligned to the alveolar margin of the bone (described above) that probably represent the exits of the cutaneous branches of the inferior alveolar nerve [65] (Fig. 3F: nvf; Fig. 9B: nf). In addition, another large and oval foramen is located on the lateroventral surface of the bone, resembling the condition in *Proterosuchus fergusi* (BSPG 1934-VIII-514; RC 59, 96; SAM-PK-K10603) and a referred specimen of *Archosaurus rossicus* (PIN 1100/78). The oval posterior “foramen” described by Thulborn ([20]: fig. 4b) is probably an artefact resulting from sediment covering the bone. The medial surface of the dentary is widely exposed on the right element. The symphysis seems to be restricted to the most anterior end (Fig. 3H: sy), but the limits of the facet cannot be confidently assessed due to poor preservation. The Meckelian canal extends along most of the medial surface of the dentary and is situated close to the mid-height of the bone (Fig. 3H: Mc; Fig. 9A: Mc), as is the case in “*Chasmatosaurus*” *yuani* (IVPP V90002), a dentary referred to *Archosaurus* (PIN 1100/78), *Sarmatosuchus otschevi* [51], *Erythrosuchus africanus* (NHMUK R2790) and *Shansisuchus shansisuchus* [46]. The Meckelian canal tapers anteriorly, reaching close to the anterior margin of the dentary and probably approaching the symphyseal facet. The canal becomes more clearly defined at its dorsal and ventral margins towards its posterior end.

The right dentary preserves two partial crowns in situ at its anterior end (Fig. 3H). The anterior end of the left dentary preserves four poorly preserved teeth in situ (Figs. 3F, 9B) and the most complete portion of the left dentary possesses five teeth in situ with an estimated total of 17 tooth positions (Fig. 9C). Accordingly, it can be assumed that the dentary tooth count exceeded 22 positions (Fig. 5A), in agreement with the tooth count estimated for the maxilla, and resembling the condition in *Protorosaurus speneri* [4], *Prolacerta broomi* [50], [59], medium to large-sized specimens of *Proterosuchus fergusi* (BSPG 1934-VIII-514; GHG 231; SAM-PK-11208; RC 96) and “*Chasmatosaurus*” *yuani* (IVPP V90002). By contrast, in *Shansisuchus shansisuchus* [46], *Sarmatosuchus otschevi* [51], *Garjainia prima* (PIN 2394/5) and *Euparkeria capensis* [48] the dentary tooth count is lower than 20. Although the available dentary tooth crowns of *Tasmaniosaurus triassicus* are very poorly preserved, their morphology agrees with that of the maxillary teeth.

#### Splenial

The left splenial is preserved next to the right dentary and lacks its anterior end (Figs. 3G, 9A; Table 4). As noted above, the interpretation of Camp & Banks [19] that the bone represents a splenial rather than a dentary is followed (contra Thulborn [20]). Indeed, the overall morphology of the bone is almost identical to that of the splenial of *Proterosuchus fergusi* (BSPG 1934-VIII-514). The bone is interpreted as being exposed in lateral view because of the presence of a thick, rounded tuberosity that extends longitudinally next to the ventral margin of the bone (Fig. 3G: vt; Fig. 9A: vt). This tuberosity is observed on the lateral surface of the splenial of other archosauromorphs and delimits the ventral margin of the Meckelian canal (e.g. *Prolacerta broomi*: BP/1/2675; *Erythrosuchus africanus*: [53], fig. 16d), indicating that the preserved splenial of *Tasmaniosaurus triassicus* is a left element. The most anterior preserved portion of the splenial possesses a planar lateral surface that becomes concave posteriorly, extending along most of the length of the bone, representing the medial wall of the Meckelian canal (Fig. 3G: Mc; Fig. 9A: Mc). As a result, the ventral border of the splenial is transversely thicker than the laminar dorsal margin of the bone. The splenial of *Tasmaniosaurus triassicus* possesses a long and posteriorly tapering posterior process (Fig. 3G: pp; Fig. 9A: pp), which probably lacks its distal end, and should have articulated with the angular and prearticular. This condition resembles that observed in *Proterosuchus fergusi* (BSPG 1934-VIII-514; SAM-PK-K10603) and *Euparkeria capensis* (UMZC T692).

### Postcranium

#### Presacral vertebrae

Two presacral vertebrae can be identified in the holotype of *Tasmaniosaurus triassicus* [19], [20] (Fig. 10A, D). One of the vertebrae is mostly exposed in lateral view (Figs. 10A, 11E, F, I) and the other vertebra is visible in posterior view and partially in right lateral view (Figs. 10D, 11A, B) (Table 5). The latter vertebra possesses a diapophysis that it is situated immediately above the neurocentral boundary (Fig. 10D: di; Fig. 11B: di). A thick lamina extends ventrally from the base of the diapophysis (Fig. 10D: tl; Fig. 11A, B: tl), closely resembling the condition observed in the cervico-dorsal (i.e. posterior cervical to anterior dorsal) vertebrae of *Prolacerta broomi* (BP/1/2675) and *Proterosuchus fergusi* (NM QR 1484; SAM-PK-11208). In the case of *Prolacerta broomi* and *Proterosuchus fergusi* this thick lamina hosts an articular facet for a third head of the associated rib, but this condition cannot be assessed in *Tasmaniosaurus triassicus* because the relevant area is covered by matrix. However, the presence of this thick lamina suggests that the vertebra mostly exposed in posterior view belongs to the cervico-dorsal transition region. The vertebra that it is mostly visible in lateral view is an anterior or middle dorsal vertebra because an anteroventrally oriented paradiapophyseal lamina extends towards the anterodorsal corner of the centrum (Fig. 10A: pdl; Fig. 11E: pdl), as occurs in the anterior and middle dorsal vertebrae of other archosauromorphs (e.g. *Tanystropheus longobardicus*: PIMUZ T2817; *Spinosuchus caseanus*: [66]; *Prolacerta broomi*: BP/1/2675; *Erythrosuchus africanus*: [53]). By contrast, in more posterior dorsal vertebrae the paradiapophyseal or anterior centrodiapophyseal lamina is more vertical or both articular facets are merged with one another.

The cervico-dorsal vertebra of *Tasmaniosaurus triassicus* possesses a non-notochordal centrum that it is slightly transversely compressed at mid-length (Figs. 10D, 11A, B), resembling the condition in the vast majority of archosauromorphs [67]. The posterior articular surface is subcircular and moderately concave (Fig. 10D: pf; Fig. 11A, B: pf), but the collapse of cortical bone on this articular surface exaggerates the degree of concavity. The lateral surface of the centrum possesses a shallow and not well-defined lateral fossa, as also occurs in other basal archosauromorphs (e.g. *Proterosuchus fergusi*: SAM-PK-11208; *Erythrosuchus africanus*: NHMUK R3592; *Euparkeria capensis*: UMZC T692j; *Cuyosuchus reigi*: MCNAM 2669; *Tarjadia ruthae*: [68]) and crown archosaurs (e.g. *Pseudopalatus buceros*: [69]; *Arizonasaurus babbitti*: [70]; *Aetosauroides scagliai*: [71]; *Marasuchus lilloensis*: PVL 3870; *Pantydraco caducus*: [72]). By contrast, in the basal archosauriform *Koilamasuchus gonzalezdiazi* the lateral fossa of the dorsal vertebrae is deeper and better defined [73]. The posteroventral border of the centrum is damaged and, as a result, the presence or absence of bevelling for reception of an intercentrum cannot be assessed. The neurocentral suture is not visible in this vertebra, but this may reflect poor preservation and the layer of lacquer that covers the relevant area.

The neural arch of the cervico-dorsal vertebra of *Tasmaniosaurus triassicus* is proportionally tall when compared with the height of the centrum. Indeed, the height of the neural arch up to the base of the neural spine is subequal to the height of the centrum, resembling the condition in *Proterosuchus fergusi* (SAM-PK-11208) and *Euparkeria capensis* (SAM-PK-5867). By contrast, the height of this region of the neural arch is proportionately lower in *Prolacerta broomi* (BP/1/2675). The neural canal is trapezoidal in posterior view, being considerably wider than tall (Fig. 11A, B: nc). The width of the neural arch immediately dorsal to the level of the neural canal is lower than that at level of the neurocentral suture. The diapophysis is situated well ventrally on the neural arch and mainly laterally directed in posterior view, but with a small ventral component. The distal end of the diapophysis is missing and, as a result, its articular facet is not preserved. There is no evidence of a posterior centrodiapophyseal lamina, but this may be a consequence of the poor preservation of the bone. The right postzygapophysis is the best preserved and possesses a lateroventrally facing articular facet (Fig. 10D: poz; Fig. 11B: poz). Both postzygapohyses lack their posterior ends and, as a result, it is not possible to assess the presence of a post-spinal fossa or of a hyposphene. A thin and sharp lamina connects the right postzygapophysis with the base of the neural spine, representing a possible spinopostzygapophyseal lamina (Fig. 10D: spl; Fig. 11B: spl), as also occurs in the trilophosaurids *Trilophosaurus* and *Spinosuchus caseanus* [66].

The neural spine lacks most of its posterior margin and its distal end. The preserved portion of the neural spine is as tall as the posterior end of the centrum, indicating that when complete the spine would have been subequal in height to or taller than the centrum. This condition resembles that of *Proterosuchus fergusi* (SAM-PK-11208), but contrasts with neural spines that are shorter than the centrum of the cervico-dorsal vertebrae of *Prolacerta broomi* (BP/1/2675). The neural spine seems to have a narrow transverse expansion at its most distally preserved tip, which is asymmetric in posterior view and is likely a preservational artefact (Fig. 10D: de; Fig. 11B: de). Due to the absence of the distal tip of the neural spine it is not possible to assess the presence or absence of a spine table.

The anterior or middle dorsal vertebra of *Tasmaniosaurus triassicus* lacks part of the distal margin of the neural spine and most of the postzygapophyses (Figs. 10A, 11E, F, I). The centrum is moderately transversely compressed at a point slightly anterior to mid-length. The lateral surface of the centrum possesses a shallow and not well-defined lateral fossa situated immediately below the level of the neurocentral boundary (Fig. 10A: ld), resembling the condition present in the cervico-dorsal vertebra. The morphology of the anterior surface of the centrum is not observable because it is covered with matrix, but the posterior articular surface of the centrum is moderately concave and oval, being taller than wide (Fig. 11F, I: pf). The ventral surface of the centrum is mostly exposed and is continuously convex, without any keel or groove (Fig. 11I). The neurocentral suture cannot be observed, as is the case in the other presacral vertebra (see above), but this is also likely because of the poor preservation of the element combined with the lacquer layer that covers its surface. Although there is no clear bevelling of the anterior and posterior margins of the centrum, the presence or absence of intercentra in the dorsal vertebrae of *Tasmaniosaurus triassicus* cannot be properly assessed.

Only the base of the diapophysis is preserved and is situated level with the mid-length of the centrum. The neural arch possesses a paradiapophyseal lamina (Figs. 10A: pdl; Fig. 11E: pdl), as described above. This lamina reaches the anterodorsal corner of the centrum, at which point the base of a parapophysis appears to be present. However, the area is rather damaged and the presence of a parapophysis cannot be assessed with certainty. The posterior centrodiapophyseal lamina is absent, as is also the case in the basal neodiapsid *Youngina capensis* (BP/1/3859) and the archosauromorphs *Prolacerta broomi* (BP/1/2675) and *Proterosuchus fergusi* (SAM-PK-K140; GHG 363), in which only a paradiapophyseal or anterior centrodiapophyseal lamina is present below the diapophysis. A well-developed and thin prezygodiapophyseal lamina extends from the base of the diapophysis to the base of the prezygapophysis (Fig. 10A: prdl; Fig. 11E: prdl), as also occurs in the enigmatic neodiapsid *Helveticosaurus zollingeri* (PIMUZ T4352), the basal archosauromorphs *Tanystropheus longobardicus* ([74]: fig. 52–54), *Trilophosaurus* and *Spinosuchus caseanus* [66]; *Protorosaurus speneri* (BSPG 1995-I-5 cast of WMSN P47361), *Macrocnemus bassanii* (PIMUZ T2472, 4822), *Prolacerta broomi* (BP/1/2675), *Erythrosuchus africanus* (NHMUK R3592; [53]), *Shansisuchus shansisuchus* ([46]: fig. 21), *Euparkeria capensis* (UMZC T921), and several crown archosaurs (e.g. *Hypselorhachis mirabilis*: [75]; *Silesaurus opolensis*: [76]; *Herrerasaurus ischigualastensis*: PVSJ 373, [77]). By contrast, *Proterosuchus fergusi* (SAM-PK-K140; GHG 363) lacks a prezygodiapophyseal lamina on the neural arch of the dorsal vertebrae. The exposed right prezygapophysis is moderately long and mainly anteriorly directed, but with a small dorsal component (Fig. 10A: prz; Fig. 11E: prz). The right postzygapophysis is completely lost, but the left postzygapophysis is exposed in medial view (Fig. 10A: poz; Fig. 11E: poz). The postzygapophysis is posteriorly extended beyond the posterior margin of the centrum.

The neural arch possesses a moderately deep depression immediately lateral to the base of the neural spine, situated level with the mid-length of the base of the diapophysis. Similar depressions are observed in the dorsal vertebrae of *Protorosaurus speneri* (BSPG 1995-I-5), *Mesosuchus browni* (SAM-PK-6046), *Prolacerta broomi* (BP/1/2675), *Proterosuchus fergusi* (GHG 231) and *Erythrosuchus africanus* (NHMUK R3592), although in the above mentioned species they are considerably deeper. The base of the neural spine is well extended anteroposteriorly along most of the length of the neural arch. The neural spine is mainly dorsally directed, but with a distinct posterior component (Fig. 10A: ns; Fig. 11E: ns), resembling the condition observed in the anterior and middle dorsal vertebrae of *Proterosuchus fergusi* (SAM-PK-11208), *Shansisuchus shansisuchus* [46] and *Erythrosuchus africanus* (NHMUK R3592). The distal end of the neural spine lacks a distinct transverse expansion, resembling the condition present in the anterior and middle dorsal vertebrae of most specimens of *Proterosuchus fergusi* (GHG 231, SAM-PK-11208; except in the holotype of “*Chasmatosaurus alexandri*”: NM QR 1482) and *Erythrosuchus africanus* (NHMUK R3592), and the middle dorsal vertebrae of *Prolacerta broomi* (BP/1/2675). The posterior margin of the neural spine is slightly concave along its distal half in lateral view, resulting in a pointed posterodorsal corner. The anterior margin of the neural spine is mostly straight but possesses a low and rounded anterior projection at its distal end. As a result, the neural spine of the anterior or middle dorsal vertebra of *Tasmaniosaurus triassicus* is anteroposteriorly longer distally than it is immediately above the level of the zygapophyses, resembling the condition of *Protorosaurus speneri* (BSPG 1995-I-5), *Proterosuchus fergusi* (GHG 363) and *Erythrosuchus africanus* (NHMUK R3592). By contrast, in *Prolacerta broomi* (BP/1/2675) the anterior and middle dorsal neural spines are sub-rectangular in lateral view.

Three possible disarticulated intercentra are preserved close to the interclavicle (Fig. 11J; Fig. 13: ic). These bones are oval to pentagonal in outline, being wider (6.5 mm) than tall (5.1 mm). The shape of these bones closely resembles those of other archosauriform intercentra (e.g. *Proterosuchus fergusi*: NM QR 1484; SAM-PK-11208) and the ratio between centrum and intercentrum width is approximately 1.8 in *Tasmaniosaurus triassicus*, resembling the ratio observed in the dorsal vertebrae of *Proterosuchus fergusi* (e.g. SAM-PK-11208: ratio 1.6). It cannot be determined whether the possible intercentra belong to the cervical, dorsal or proximal caudal series.

#### Presacral ribs

Multiple partial bones of the holotype of *Tasmaniosaurus triassicus* probably represent fragmentary cervical or dorsal ribs [20] (Fig. 7A: dr; Fig. 11C, D: dr, G, H; Fig. 14B: dr; Fig. 17C). For example, the rod-like fragment of bone preserved next to the skull roof that Camp & Banks [19] interpreted as a partial postorbital is probably a fragment of rib shaft (Fig. 7A: dr). This bone is too large to represent the ventral process of a postorbital and too robust to be either the anterior or posterior process of a postorbital. The morphology of this bone resembles that of a dorsal rib shaft [19], including the presence of a longitudinal sulcus that is usually present on the posterior surface of dorsal ribs. The probable anterior surface of this dorsal rib shaft is preserved as a natural mould. This surface possesses a longitudinal tuberosity slightly anteriorly displaced from the mid-width of the shaft.

Camp & Banks [19] identified a probable partial scapula, but this fragment of bone is instead possibly assignable to the proximal end of a dorsal rib that is not very informative (cf. Thulborn [20]) (Fig. 11G). The putative parietal that Thulborn [20] interpreted as enclosing a supratemporal fenestra is reidentified here as the proximal end of a rib (Fig. 17C). The rib head is dichocephalous, with well-developed capitulum and tuberculum (Fig. 17C: ca, tu). The exposed surface of the rib shaft is continuously convex and, as a result, it is probably preserved in anterior view. It cannot be determined if this rib belonged to the posterior cervical or dorsal series.

In the same block that preserves the anterior or middle dorsal vertebra there is a fairly complete dorsal rib with a preserved length of 101.9 mm ([19]: fig. 6e) (Fig. 11C). The shaft of this rib seems to be almost complete, being preserved as fragments of bone and natural moulds. The shaft is rod like and continuously bowed medially. There is no sharp bend between the proximal end and the shaft, contrasting with doswelliids [78]–[80], but resembling the condition observed in the vast majority of archosauromorphs [73]. The proximal end of the rib is poorly preserved and only a single probable capitulum is recognizable, but an assessment as to whether the rib was holocephalous or dichocephalous is not possible.

Three well-preserved dorsal rib shafts are preserved close to each other in the same block that contains the right lacrimal, with the largest of these rib shafts having a maximum preserved length of 130.8 mm (Fig. 11H). These ribs possess the same morphology as the elements described above. Another large fragment of probable dorsal rib shaft is preserved next to the non-*Tasmaniosaurus* tiny maxilla (see below), but does not provide further information.

#### Gastralia

Thulborn ([20]: 133, 135) provided a detailed and accurate description of the gastralia of *Tasmaniosaurus triassicus*. He identified three types of gastralia, in agreement with the different types of gastralia found in *Proterosuchus fergusi* (NM QR 1484; [20]). First, V-shaped elements that resemble the gastralia found in the anterior half of the trunk region (Fig. 12B: ga); second, long, slender and rod-like elements that are curved and tapered at one end (Fig. 14A: ga), which correspond to the gastralia found at about mid-length of the trunk region with the curved extremities extended upwards and posteriorly onto the flank of the animal; and finally, broad U-shaped elements that are found in the posterior half of the trunk region (Fig. 11D: ga, K).

#### Caudal vertebrae

Fourteen to sixteen caudal vertebrae can be identified in the holotype of *Tasmaniosaurus triassicus* (Figs. 10B, 12; Table 6). Four middle caudal vertebrae are preserved in articulation with each other, with the most posterior one lacking its posterior half (Figs. 10B, 12A). Another sequence of seven articulated and badly preserved middle or distal caudal vertebrae is preserved, with the sequence gently bowed dorsally along its length, with one of the vertebrae represented only by a fragment of centrum (cf. Thulborn, [20]) (Fig. 12B). Camp & Banks [19] originally described thirteen caudal vertebrae in this sequence, but as discussed by Thulborn [20] those authors probably misinterpreted some fractures as the ends of centra. Three or four additional caudal vertebrae are present but poorly preserved a few centimetres above the sequence of seven articulated vertebrae (cf. Thulborn [20]) (Fig. 12C). Indeed, these vertebrae may represent the continuation of the latter sequence because the two sequences are aligned with each other in the same block and have the same degree of dorsal bowing. It seems that no vertebra is missing between the two series because the gap between them as preserved would have been filled by the estimated missing length of the partial centra at the ends of each sequence.

The sequence of four middle caudal vertebrae is exposed in lateral view and poorly preserved (Figs. 10B, 12A). The anterior end of the sequence can be recognised due to the posterior orientation of the haemal arches (Fig. 10B: ha; Fig. 12A: ha). The zygapophyses are weakly anteroposteriorly developed and horizontally oriented with respect to the longitudinal axis of the tail. There is no clear evidence of transverse processes, but it is likely that this is a consequence of the poor preservation of the bones. Part of the base of a neural spine is preserved on the second vertebra of the series, and seems to have been anteroposteriorly long (Fig. 10B: ns; Fig. 12A: ns). In the other vertebrae the neural spines are completely missing (if they were actually present in life).

The sequence of seven middle or distal caudal vertebrae is badly preserved (Fig. 12B). The widest vertebrae of the sequence belong to the most anterior elements, as is also suggested by the orientation of a probable haemal arch between the first and second elements of the series. The first two vertebrae are represented by partially exposed centra and the more posterior vertebrae seem to lack their centra and the bases of the neural arches are exposed, with the rest of the neural arch covered by the matrix. Indeed, the base of a transverse process seems to be visible in ventral view on the fifth vertebra of the sequence (Fig. 12B: tp). In none of the vertebrae of this series were zygapophyses or neural spines recognized. The second sequence of three or four vertebrae likely represents the vertebrae immediately proximal to the sequence of seven vertebrae described above (Fig. 12C). These vertebrae are very poorly preserved and seem to be preserved mixed together with other fragments of bone, one of which was originally interpreted as part of an ilium by Camp & Banks [19]. The morphology of these vertebrae is congruent with that of the seven articulated vertebrae and no further information can be provided. A few centimetres to the right of the above-described series, but belonging to a different block that has likely been artificially assembled in its current position, is preserved a probable anterior or middle caudal vertebral centrum (Fig. 12D). A few millimetres above this probable vertebra there is a plate-like bone that may belong to a large neural spine. However, these bones are very poorly preserved and they should be considered as indeterminate elements.

#### Haemal arches

Most of the information of the haemal arches of *Tasmaniosaurus triassicus* comes from an element exposed in transverse section (Fig. 10E; Fig. 12C: ha) and an almost complete chevron exposed in right lateral view (Fig. 10F; Fig. 12D: ha) (Table 7). The haemal arch exposed in transverse section is subtriangular and lies in the same block as the series of seven middle or distal caudal vertebrae. The haemal canal is closed dorsally and has an oval outline, being considerably taller than wide (Fig. 10E: hc; Fig. 12C: hc). The proximal end of the chevron lacks the low lateral expansions observed in *Koilamasuchus gonzalezdiazi* [73], but this is probably a preservational artefact. The ventral half of the haemal arch tapers gradually ventrally, but lacks its distal end. The haemal arch preserved in right lateral view lacks the proximal end and lies a few centimetres to the right of the above-described haemal arch, but in a different block. The lateral surface of the haemal arch is moderately convex on the proximal preserved portion of the bone, which should be at the level of the haemal canal. The lateral surface of the bone becomes planar ventrally. The distal end of the haemal arch is anteroposteriorly expanded, resulting in a plate-like structure (Fig. 10F: pdp; Fig. 12D: pdp), resembling the condition observed in the tail of *Proterosuchus fergusi* (NM QR 1484).

Fragments of three haemal arches are preserved in articulation with their respective centra in the sequence of four middle caudal vertebrae (Fig. 10B: ha; Fig. 12A: ha). The haemal arches are moderately long, but their total lengthes cannot be determined. Additionally, the remains of one or two haemal arches are preserved in the proximal region of the sequence of seven articulated middle or distal caudal vertebrae. However, these bones are poorly preserved and not informative.

#### Interclavicle

The interclavicle is probably the element with the least controversial identification among the bones of the holotype of *Tasmaniosaurus triassicus* [19], [20]. The interclavicle is exposed in dorsal view and is almost complete, only lacking portions of its anterior margin (Fig. 13; Table 8). The dorsal surface of the interclavicle is continuously concave, as is also observed in other archosauromorphs (e.g. *Trilophosaurus buettneri*: [55]; *Proterosuchus fergusi*: GHG 363; NM QR 1484; *Garjainia prima*: PIN 2394/5). The transition between the anterior end and the posterior process is gradual, resulting in a diamond-shaped anterior end of the interclavicle, resembling the condition of *Macrocnemus bassanii* (PIMUZ T4355), *Protorosaurus speneri* [4] and *Prolacerta broomi* (BP/1/2675). In *Garjainia prima* the transition between the anterior end and the posterior process is also gradual, but due to the lack of preservation of most of the lateral processes it cannot be assessed whether or not the anterior end of the bone was diamond-shaped (PIN 2394/5). By contrast, in *Proterosuchus fergusi* (GHG 363; NM QR 1484), *Trilophosaurus buettneri* [55] and *Mesosuchus browni* [1] the interclavicle has a characteristic T-shape morphology in dorsal view, which results from the sharp distinction between the lateral process of the anterior end and the posterior process of the bone.

The anterior end of the interclavicle is divided into two planar to slightly convex dorsal surfaces by a thin and shallow median groove (Fig. 13: md). The lateral processes are well developed laterally (Fig. 13: lp). These processes possess a straight anterior margin and a slightly concave posterior one. The anterior margin of the interclavicle is gently concave at mid-width, indicating the presence of a low median notch (Fig. 13: mn), resembling the condition of *Proterosuchus fergusi* in which the anterior median notch is also present (GHG 363) By contrast, in *Macrocnemus bassanii* (PIMUZ T4355), *Prolacerta broomi* (BP/1/2675) and *Mesosuchus browni* [1] the median notch is proportionally deeper, whereas it is absent in *Protorosaurus speneri* [4] and *Trilophosaurus buettneri* [55].

The posterior process of the interclavicle is anteroposteriorly very long and transversely narrow (Fig. 13: pp). Indeed, the width of the posterior process at mid-length is approximately 0.12 of the maximum width of the anterior end of the bone in *Tasmaniosaurus triassicus*, resembling the condition observed in *Macrocnemus bassanii* (PIMUZ T4355) and *Prolacerta broomi* (ratio approximately 0.14 in BP/1/2675). By contrast, *Proterosuchus fergusi* (GHG 363, NM QR 1484: ratio approximately 0.25–0.36), *Protorosaurus speneri* [4], *Trilophosaurus buettneri* [55], *Mesosuchus browni* (SAM-PK-6536) and *Garjainia prima* (PIN 2394/5) possess a distinctly more robust posterior process of the interclavicle. The posterior process has its strongest transverse constriction immediately posterior to the anterior end of the bone and gradually expands transversely towards the posterior tip of the interclavicle. As a result, the posterior three-quarters of the process possess a clear transverse expansion (Fig. 13: te), resembling the condition observed in rhynchosaurs (e.g. *Mesosuchus browni*; [1]), *Trilophosaurus buettneri* [55], *Prolacerta broomi* (BP/1/2675) and *Euparkeria capensis* (SAM-PK-5867). By contrast, in *Garjainia triplicostata* [81] and *Garjainia prima* (PIN 2394/5) the transverse expansion is considerably more strongly developed. In *Proterosuchus fergusi* (NM QR 1484) the posterior process of the interclavicle has parallel lateral margins without a transverse expansion. The process decreases slightly in width posteriorly, and the posterior margin of the bone, which seems to be natural, ends in a square outline, as also occurs in *Prolacerta broomi* (BP/1/2675), *Mesosuchus browni* [1] and some specimens of *Proterosuchus fergusi* (GHG 363). The dorsal surface of the posterior process possesses some shallow and narrow longitudinal grooves.

#### Femur?

Camp & Banks ([19]: fig. 6j) interpreted a long bone that partially overlaps a tibia, and which is preserved in the same block as the middle caudal vertebrae, as a fibula. However, Thulborn [20] identified this bone as a probable left femur because it was at least as long and broad as the tibia and remnants of the fibula may lie alongside the distal end of the tibia. The observations of Thulborn [20] are here supported because this bone is approximately 112% of the length of the tibia and at least one of its ends is missing (Fig. 14A: ?fe; Table 9). The putative femoral shaft seems to be slightly narrower than that of the tibia, which would argue against this identification. However, this condition may be a consequence of post-mortem deformation, as is also seen in other bones of the specimen (e.g. one of the tibiae, see below), and/or that the femoral shaft is preserved in medial or lateral view. An alternative explanation that would maintain the original identification of this bone as a fibula would be that the distal end of the tibia, which is preserved as a natural mould, is broken off. Nevertheless, the distal margin of the mould is smooth and well defined, suggesting that the entire length of the tibia is preserved. Accordingly, the currently available evidence favours the identification of the bone as a partial femur, but this interpretation should be considered tentative (cf. Thulborn [20]). The shaft of this bone is poorly preserved and neither of its ends can be properly identified, and thus no useful anatomical information is available.

#### Tibia

A large long bone is preserved a few centimetres to the right of and in the same block as the sequence of four middle caudal vertebrae (Fig. 10C; Fig. 14A: t; Table 9). Both Camp & Banks [19] and Thulborn [20] identified this bone as a tibia. Indeed, the morphology of the bone is very similar to that of the tibia of a South African proterosuchid (NM QR 880) and “*Chasmatosaurus*” *yuani* (IVPP V2719) and the identification of previous authors is thus followed here. However, the well-expanded end identified as the distal end by Camp & Banks ([19]: fig. 6j) is reinterpreted here as the proximal end. Another bone that is very similar in size and shape is preserved a few centimetres above the skull roof (Fig. 14B). Camp & Banks [19] identified this bone as the other tibia, but Thulborn [20] suggested that there was not enough evidence to support that interpretation and considered it instead as an indeterminate limb bone. Based on the extremely similar morphology of both bones, for example in the degree of asymmetry of the proximal end, it is here considered that the original interpretation of this bone as the opposite tibia is very likely (cf. Camp & Banks [19]).

The tibiae are very strongly compressed due to post-mortem taphonomic modifications, as is the case in several bones of the specimen. The proximal end of the bone is asymmetric, with the proximal expansion more strongly developed in one direction than the other. The more strongly developed expansion should correspond to the ventral ( =  posterior surface in a cursorial animal in which limbs are orientated vertically) condyle of the bone (Fig. 10C: vc; Fig. 14: vc), as is the case in *Prolacerta broomi* (BP/1/2676), proterosuchids (e.g. NM QR 880, IVPP V2719) and *Erythrosuchus africanus* [53]. It is not possible to identify which side each of the tibiae is from because their surfaces have been crushed and distorted by the strong transverse compression of the bones. Nevertheless, both tibiae should be exposed in different views (i.e. in lateral and medial views, respectively) because the posterior condyle of the bone is preserved on the left side in both elements. In the tibia directly associated with the probable femur the proximal end and part of the shaft are preserved, whereas the distal third of the bone is mostly preserved as a natural mould (Fig. 10C; Fig. 14A: mde). The other tibia is more extensively damaged and lacks its distal end.

The tibia of *Tasmaniosaurus triassicus* is a rather robust bone, as is the case in a South African proterosuchid (NM QR 880), but contrasting with the considerably more gracile tibia of *Prolacerta broomi* (BP/1/2676). The proximal surface of the tibia possesses two distinct proximal convexities that are separated by a transverse concavity, resembling the condition of *Prolacerta broomi* (BP/1/2676) and proterosuchids (NM QR 880, IVPP V2719). This depression divides the proximal surface of the tibia into a shorter dorsal portion that corresponds to the cnemial crest (Fig. 10: cc; Fig. 14: cc) and a longer ventral condyle in lateral view (Fig. 10: vc; Fig. 14: vc). Both dorsal and ventral margins of the proximal end of the tibia are rounded in side view. The shaft is slightly posteriorly bowed and lacks the large and deep pit described for *Erythrosuchus africanus* [53], resembling instead the condition observed in *Prolacerta broomi* (BP/1/2676). The distal end of the bone possesses a shallow, poorly defined longitudinal groove along its lateral or medial surface. However, this feature may be an artefact due to breakage following the strong transverse compression suffered by the bone. The distal end is moderately dorsoventrally expanded, resembling the condition in *Prolacerta broomi* (BP/1/2676), “*Chasmatosaurus*” *yuani* (IVPP V2719), *Shansisuchus shansisuchus* [46] and *Euparkeria capensis* [48]. By contrast, the degree of expansion of the distal end of the tibia is proportionally larger in *Erythrosuchus africanus* (NHMUK R3592).

#### Foot

Two different clusters of autopodial bones are preserved in the holotype of *Tasmaniosaurus triassicus* (Figs. 15, 16; Table 10). One of the groups, which is preserved in the same block as the complete tibia, includes a hook-shaped fifth metatarsal [19] and, as a result, is interpreted as a disarticulated partial foot (Fig. 15). Thulborn [20] interpreted the other group of autopodial bones (Fig. 16) as belonging to the manus. However, the preserved ungual phalanx is poorly curved (Fig. 10L; Fig. 16: un), resembling the condition observed in the foot of *Proterosuchus fergusi* (SAM-PK-K140). By contrast, the manual unguals of *Proterosuchus fergusi* are more strongly ventrally curved than the pedal claws (SAM-PK-K140). Accordingly, this group of bones is also interpreted as a partial foot, but this interpretation should be considered tentative.

In the group of bones that lie in the same block as the complete tibia (Fig. 15), Thulborn [20] identified the presence of all the elements of the metatarsus (i.e. from metatarsal I to V). However, the elements that Thulborn interpreted as metatarsals II and III seem to belong to a single, compressed indeterminate metatarsal (Fig. 15: mtt) with a length of 23.1 mm and a width of 14.5 mm. The bone interpreted by Thulborn [20] as metatarsal I is poorly preserved, but seems to be more gracile than metatarsal I of *Proterosuchus fergusi* (SAM-PK-K140). This element in *Tasmaniosaurus triassicus* may alternatively belong to a proximal phalanx (Fig. 15: ?pph). A proximal end of a phalanx is preserved in articulation with this bone, in agreement with the interpretation of Thulborn ([20]: fig. 12).

The bone identified by Thulborn [20] as metatarsal V does not possess the typical hook-shaped proximal end observed in other archosauromorph fifth metatarsals (e.g. [82], [83]) and a fifth metatarsal is instead recognized one centimetre below this bone (cf. Camp & Banks [19]). The former bone preserves its proximal end and distal half, but lacks part of the shaft that is available as a natural mould. The length of the bone in comparison with that of metatarsal V suggests that it is probably a metatarsal II (Figs. 10G, 15: ?mttII), using the foot of *Proterosuchus fergusi* for comparison (SAM-PK-K140; [82]: fig. 10). The shaft of the bone is straight and the proximal and distal ends are sub-equally expanded, as occurs in *Proterosuchus fergusi* (SAM-PK-K140). The proximal end of the bone is partially covered by matrix and the distal end possesses a non-ginglymoideal articular surface. A poorly preserved, partial bone aligned with the probable metatarsal II and indicated by Thulborn ([20]: fig. 12) mostly with a dotted line seems to correspond to another metatarsal, probably metatarsal I or III given its position. The bone identified by Thulborn [20] as metatarsal IV does indeed seem to correspond to a metatarsal due to its block-shaped end. This element likely represents either a third or fourth metatarsal (Fig. 15: mtt).

Thulborn ([20]: fig. 12) labelled the metatarsal V as the probable pubis of Camp & Banks [19] and considered it an unidentifiable fragment of bone. However, it seems that the latter authors correctly identified this bone as a fifth metatarsal based on their description and figure (Fig. 15: mttV). The metatarsal V of *Tasmaniosaurus triassicus* possesses a hook-shaped proximal end [19] (Fig. 10H: hpe; Fig. 15: hpe), resembling the condition widespread among basal diapsids (e.g. *Gephyrosaurus bridensis*: [84]; *Macrocnemus bassanii*: PIMUZ T4355; *Prolacerta broomi*: BP/1/2676; *Proterosuchus fergusi*: SAM-PK-K140; *Erythrosuchus africanus*: BP/1/2096). It is not possible to discern if the fifth metatarsal of *Tasmaniosaurus triassicus* is exposed in dorsal or ventral view because no lateral or medial tubercle can be recognised. The shaft of the metatarsal is short and the distal end is damaged.

Five small bones are preserved next to the probable metatarsal II and as proposed by Thulborn [20] these are probably pedal phalanges (Fig. 16: ph). Four of these bones are preserved close to the distal end of the probable second metatarsal and the fifth one is preserved next to the shaft of the metatarsal. The two superimposed phalanges identified by Thulborn ([20]: fig. 12) seem to actually correspond to a single element. Only two of these bones seem to be complete and they probably represent small distal non-ungual phalanges. In none of these bones can a collateral pit be observed, but this is probably due to poor preservation.

Next to the above-described autopodial elements there are preserved two small and badly preserved bones (Fig. 15: spt). Camp & Banks [19] identified these disc-like elements, one preserved as bone and the other as a natural mould, as tarsal elements. The largest linear dimensions of these bones are 14.5 mm and 10.3 mm, respectively. The position and size of the bones would suggest that they are distal tarsals, but they are essentially featureless. Accordingly, they should be considered indeterminate bones, in agreement with Thulborn [20].

Among the other group of pedal autopodial elements there is preserved a bone with a distinct trochlea that possesses a large, circular collateral pit (unidentified “x” fragments of Thulborn [20]: fig. 11]) (Fig. 10I: clp; Fig. 16: clp). Collateral pits are present in the non-ungual phalanges of *Proterosuchus fergusi*, but not in the metatarsals (SAM-PK-K140). Thus, this bone probably represents a large proximal pedal phalanx exposed in side view (Fig. 16: pph). The distal trochlea is circular (Fig. 10I: dtr) and the well-defined collateral pit occupies most of its surface. It cannot be assessed if the distal end was ginglymoideal because only either the lateral or medial surface is exposed. The shaft of the phalanx (Fig. 16: sh) is severely damaged and probably preserved in two parts, but it seems to have been considerably dorsoventrally lower than the trochlea and proximodistally long. Close to this proximal phalanx there are four bones roughly aligned to each other (Fig. 13: ab, ph, pph). One of the elements is a stout phalanx exposed in ventral view (Fig. 10J; Fig. 16: ph). It has a distinctly ginglymoid distal end with shallow and poorly defined collateral pits (Fig. 10J: clp, dtr). The proximal end of the bone is more transversely expanded than the distal end. Next to this phalanx lies a fragment of metatarsal (Fig. 16: mtt), identified as such because it possesses a moderately convex articular end with a shallow depression on the surface of the shaft immediately below the articular end. This element should represent a second, third or fourth metatarsal. The third bone possesses a ginglymoideal distal trochlea, indicating that it should belong to a proximal phalanx given its size (Fig. 16: pph). Finally, the fourth bone is gracile and poorly preserved. It should represent either a metatarsal or a phalanx (Fig. 16: ab). The element labelled as a probable metacarpal impression by Thulborn ([20]: fig. 11) probably represents a poorly preserved proximal phalanx (Fig. 16: ?pph).

The pedal ungual phalanx is mostly exposed in side view and is poorly ventrally curved (Fig. 10L; Fig. 16: un). It lacks a distinct flexor tubercle, as is the case in the pedal unguals of *Proterosuchus fergusi* (SAM-PK-K140). A collateral groove is not visible in this claw, probably as a result of poor preservation.

### Problematic Bones

#### Right postorbital of Camp & Banks

The bone identified by Camp & Banks [19] as a doubtful right postorbital is a comma-shaped impression of bone in the matrix (Fig. 17A). This bone should be considered indeterminate in agreement with Thulborn [20].

#### Squamosal of Camp & Banks

A hook-shaped bone is preserved a few centimetres above the complete tibia (Fig. 17B). Camp & Banks [19] identified this bone as a probable left squamosal. However, this bone is too large and the morphology does not match with that of a squamosal preserved in dorsal view. For example, if this bone is interpreted as a squamosal it would have formed the lateral, posterior and most of the medial borders of the supratemporal fenestra, a condition not observed in any other basal archosauromorph. Thulborn [20] considered this bone as a probable gastralium, but no evidence could be recognized in this study to support that interpretation and the bone is considerably more bowed than the gastralia known in proterosuchids (NM QR 1484). On the other hand, the bone resembles in overall aspect an ectopterygoid exposed in ventral view. However the lateral process is considerably thinner than those observed in other basal archosauromorphs (e.g. *Mesosuchus browni*: [1]; proterosuchids: NM QR 880). In addition, if the bone is interpreted as an ectopterygoid it seems to be rather large in comparison with the other preserved cranial bones of *Tasmaniosaurus triassicus*. If interpreted as an ectopterygoid the bone has a maximum anteroposterior length of 29.1 mm and a transverse width of 24.3 mm. Accordingly, although the overall shape of the bone is reminiscent of an ectopterygoid, it is better to consider it as an indeterminate element for the sake of caution.

#### Supratemporal fenestra of Thulborn

In the articulated skull roof of *Tasmaniosaurus triassicus* described above are preserved both parietals in articulation with one another, which form the entire medial border of both supratemporal fenestrae (Figs. 3E, 7A). The identification by Thulborn [20] of bones (i.e. supposed parietal, squamosal, postorbital) surrounding a supposed supratemporal fenestra (Fig. 17C) is undermined by the presence of both parietals in the articulated skull roof. By contrast, the area of bone identified by Thulborn [20] as a parietal can be alternatively interpreted as the proximal end of a dichocephalous rib (see above).

#### Caudal vertebra?

In the same block that contains the lacrimal and three long dorsal rib shafts there is a very small element that resembles a vertebra exposed in anterior or posterior view. The maximum preserved height (assuming that this is indeed a vertebra) of the bone is 6.1 mm and the centrum width and height are 3.8 mm and 1.7 mm, respectively. The articular surface of the probable centrum is oval and the neural canal subequal in size to the centrum. Due to the very small size of the bone it should belong to the distal caudal series of *Tasmaniosaurus triassicus*. However, the possible neural arch has thick lateral expansions that extend well beyond the level of the centrum, contrasting with the condition expected for a caudal vertebra. Furthermore, the bone is mostly covered by matrix and lacquer and, as a result, it is considered an indeterminate element.

#### Ilium of Camp & Banks

Camp & Banks ([19]: fig. 6g) identified a possible ilium within the holotype of *Tasmaniosaurus triassicus*. Subsequently, Thulborn [20] reinterpreted the supposed ilium of Camp & Banks [19] as a series of crushed caudal vertebrae (Fig. 12C). However, it seems that Thulborn [20] misidentified the ilium of Camp & Banks [18]. Indeed, a bone almost identical in shape to that of the ilium figured by Camp & Banks [19] lies in the same block as the autopodial elements that include the ungual phalanx (Fig. 17D). This bone has a maximum linear dimension of 24.6 mm. No evidence could be identified supporting the assignment of this bone to the pelvic girdle and it is considered here as an indeterminate element.

#### Fibula of Thulborn

Thulborn [20] identified remains of a probable long bone preserved alongside the distal end of the most complete tibia as a partial fibula (Fig. 14A: ?fi). The bone possesses a maximum preserved length of 27.6 mm. The position of the bone agrees with that expected for a fibula, but it is considerably narrower than the distal end of the tibia. In *Proterosuchus fergusi* (AMNH FR 2237) the distal end of the fibula is subequal in width to that of the tibia. Alternatively, this fragment of plate-like bone may represent a partial rib shaft.

## Discussion

### Taxonomy and Phylogenetic Relationships

Camp & Banks ([19]: 149) originally diagnosed *Tasmaniosaurus triassicus* as a proterosuchid species different from other members of the group on the basis of the presence of: i) a long slightly curved premaxilla; ii) postfrontal; iii) no parietal foramen; iv) broad parietals; v) quadrate vertical; vi) palatal teeth on pterygoid and ?ectopterygoid; vii) maxillary and mandibular teeth strongly thecodont; viii) vacuity at posterior end of dentary; ix) vertebrae shallowly amphicelous; x) cervical ribs long and double-headed; xi) long hindlimbs and hindfeet; and xii) no dermal scutes. Subsequently, Thulborn [20] revisited the anatomy of *Tasmaniosaurus triassicus* and reinterpreted several of the original identifications of Camp & Banks [19] (e.g. premaxilla, skull roof, lower jaw). However, this author did not provide a formal emended diagnosis for the species. The revision of the anatomy of *Tasmaniosaurus triassicus* provided here revealed that characters (i), (v), (vii) and, in part, (vi) of the original diagnosis of the species were misinterpretations (cf. Thulborn [20]) and characters (iii), (viii) and (x) cannot be assessed due to poor preservation or the absence of the relevant elements. Characters (ii), (iv), (ix) and (xi) are widely distributed among basal archosauromorphs (e.g. *Protorosaurus speneri*, *Prolacerta broomi*, *Proterosuchus fergusi*, “*Chasmatosaurus*” *yuani*) and, as a result, they are not useful as diagnostic characters of *Tasmaniosaurus triassicus*. On the other hand, the presence of character (vi), palatal teeth on the medial margin of the pterygoid (see above), distinguishes *Tasmaniosaurus triassicus* from *Erythrosuchus africanus* [53] and *Shansisuchus shansisuchus* [46], and the absence of osteoderms contrasts with the condition observed in *Koilamasuchus gonzalezdiazi* [73], *Euparkeria capensis* [48] and more crownward archosauriforms [85]. The presence of osteoderms cannot be completely ruled out in *Tasmaniosaurus triassicus*, but their absence seems to be likely because among the multiple preserved bones of the holotype there is no evidence of osteoderms. Based on the anatomical revision of *Tasmaniosaurus triassicus* conducted here an emended diagnosis for the species is provided based on a unique combination of characters (see Systematic Palaeontology). However, it was not possible to distinguish autapomorphies for the species.

In particular, *Tasmaniosaurus triassicus* differs from non-archosauriform archosauromorphs, including *Prolacerta broomi*, in the inferred presence of a large antorbital fenestra. Regarding other putative proterosuchids, the Tasmanian species is distinct from the South African *Proterosuchus fergusi* in the presence of a posterodorsally oriented posterior process of the premaxilla, anteroposteriorly short anterior process of the maxilla, straight dorsal margin of the horizontal process (main body) of the maxilla, almost straight dentary in lateral view, and diamond-shaped anterior end and very gracile posterior process of the interclavicle (Figs. 5, 18). Furthermore, the above-mentioned cranial characters allow *Tasmaniosaurus triassicus* to be distinguished from “*Chasmatosaurus*” *yuani*, *Sarmatosuchus otschevi* and *Archosaurus rossicus* (excluding the maxillary features in comparisons to the latter two species, in which this bone is unknown). *Tasmaniosaurus triassicus* differs from the South American *Koilamasuchus gonzalezdiazi* in the presence of a posterodorsally oriented neural spine in dorsal vertebrae, the absence of a well-defined lateral fossa on the centra of the dorsal vertebrae and possibly in the absence of osteoderms.

*Tasmaniosaurus triassicus* can be also distinguished from the holotype of the Australian *Kalisuchus rewanensis* (QM F8998) in the presence of a more rounded anterior border of the antorbital fenestra, straight dorsal margin of the horizontal process of the maxilla and probably a considerably higher maxillary tooth count. *Tasmaniosaurus triassicus* differs from erythrosuchids and *Euparkeria capensis* in features previously outlined by Thulborn [20], such as the considerably higher maxillary tooth count. Accordingly, *Tasmaniosaurus triassicus* can be considered a valid species of basal archosauromorph.

Camp & Banks ([19]: 149) considered *Tasmaniosaurus triassicus* to be a member of the Proterosuchidae and closely related to the South African genus *Proterosuchus*. Furthermore, these authors discussed whether *Tasmaniosaurus* was more or less “advanced” than *Proterosuchus*. Camp & Banks [19] found some features that would make *Tasmaniosaurus* more “primitive” than *Proterosuchus*, but they also identified other traits that would support a more “advanced” position for the former taxon than *Proterosuchus*. Thulborn ([20]: 140–141) agreed with the proterosuchid affinities of *Tasmaniosaurus triassicus*, but considered the discussion about the more or less derived position of this species to be of little use because of the probable non-monophyly and poorly resolved intrarrelationships of “Proterosuchia”. This observation of Thulborn [20] was prescient, because during the late 1980s and early 1990s the first cladistic phylogenetic analyses of basal archosauromorphs found a paraphyletic “Proterosuchia”, positioning proterosuchids at the base of Archosauriformes and erythrosuchids closer to crown Archosauria ([86]–[90]; see [16] for a review of this issue). However, since then, knowledge of the phylogenetic relationships among supposed proterosuchid species or even the support for monophyly of the clade has not been substantially improved. A diagnosis of Proterosuchidae is currently problematic and recent phylogenetic analyses found that the taxonomic content of the group sensu Gower & Sennikov [51] was paraphyletic [73]. Moreover, *Tasmaniosaurus triassicus* has not yet been included in a quantitative phylogenetic analysis of basal archosauromorphs. Accordingly, the proterosuchid affinities of *Tasmaniosaurus triassicus* can be only be adequately discussed in the context of a future comprehensive phylogenetic analysis that includes multiple supposed proterosuchid and erythrosuchid species. The new anatomical information provided in this paper will help to achieve this goal.

### Olfactory Bulbs Interpretation

The latex endocast of *Tasmaniosaurus triassicus* provides reliable information on the morphology of the dorsal surface of the telencephalon, as described above (Fig. 8). However, the interpretation of the morphology of the olfactory bulbs is problematic. The olfactory tract opens anteriorly into an oval, wider than long, convex impression that includes the olfactory bulbs (Figs. 3, 7: obi). A median longitudinal groove separates the olfactory bulbs from each other (Fig. 8: lg). The impression of each olfactory bulb has a low, mostly longitudinal ridge on its ventral surface that results in distinct medial and lateral portions of each olfactory bulb impression. An almost identical feature was described for the phytosaur *Machaeroprosopus adamanensis* by Camp [91] and is also present in *Archosaurus rossicus* (PIN 1100/48) and *Sarmatosuchus otschevi* (PIN 2865/68). Camp [91] interpreted the impression medial to this ridge to be that of the olfactory bulb and the impression lateral to the ridge to be that of the vomeronasal (VN) bulb. More recently, Senter [92] revisited this interpretation and noted that crocodiles lack VN (Jacobson’s) organs but also possess the low longitudinal ventral ridge on the frontal. Senter [92] proposed an alternative interpretation that the depression lateral to the olfactory bulb was that of the ophthalmic branch of the trigeminal nerve (CN V_1_). However, Witmer ([93]: 300) noted that “the only consistent osteological correlates of the ophthalmic nerve in extant archosaurs are foramina within the premaxilla transmitting nerves carrying sensory information from the integument”. In particular, in the case of *Tasmaniosaurus triassicus* the lateral impression is wider than that of the supposed olfactory bulb (sensu Senter [92]). The presence of a nerve impression wider than that of the olfactory bulb seems unlikely and the proposed correlation of the impression with the ophthalmic nerve would contradict the observations of Witmer [93]. Accordingly, the lateral impression (Fig. 3: li; Fig. 7: li) in the area of the olfactory bulbs in *Tasmaniosaurus triassicus* has two possible interpretations: i) it belongs to a non-olfactory soft tissue (e.g. VN bulb); or ii) it is part of the olfactory bulb and indicates a considerably large olfactory system. The interpretation of the morphology of the olfactory bulbs has potentially substantial implications for the olfactory capabilities and probable mode of life of *Tasmaniosaurus triassicus*.

If the first interpretation is considered, the size and shape of the olfactory bulbs of *Tasmaniosaurus triassicus* would be very similar to those of several crown archosaurs (Table 3), including *Stagonolepis olenkae* [60], *Coelophysis bauri* (USNM 529382), *Tyrannosaurus rex* [94] and extant crocodiles (e.g. *Caiman crocodilus*: [95]). The soft tissue lateral to the olfactory bulb would correspond to a VN bulb (or other soft tissue) (Fig. 8: ?lst) and would be in agreement with the statement of Senter ([92]: 548) that the VN system was probably present in non-archosauriform archosauromorphs and proterosuchids.

It should be noted that Senter [92] inferred the absence of the vomeronasal (VN) or Jacobson’s organs in all extinct archosaurs because of its absence in extant crown archosaurs (i.e. crocodiles and birds) [93]. However, it should also be pointed out that extant archosaurs belong to groups with specialized habits and mode of life, namely a semi-aquatic habit for extant crocodiles and semi-aquatic or flying/climbing habit for birds (in particular for basal Ornithothoraces; [96], [97]). In extant non-archosaurian amniotes that returned to life in the water during evolution (e.g. aquatic turtles and mammals) the VN organs became vestigial or completely lost [98]–[100] and in those that acquired flying or climbing capabilities (e.g. arboreal lizards, catarrhini primates, chiropterans) the VN organs show high variability, including reduction and complete loss [100], [101]. On the other hand, in ground-living terrestrial amniotes the VN organs are well developed (e.g. snakes, ground-living lizards, monotremes, marsupials, rodents, ungulates, carnivores; [98], [100], [101]). As correctly noted by Senter [92], the extant phylogenetic bracket suggests that the VN system should have been absent in the most recent ancestor of crocodiles and birds. Nevertheless, it seems likely that both extant groups of archosaurs independently lost the VN organs in direct relation to their non-ground living mode of life and not as a result of inheritance of a condition present in their most recent common ancestor. Indeed, tracing the timing of the acquisition of a semi-aquatic mode of life in crocodilians and volant capabilities in ornithodirans suggest that the VN organs would have been lost in considerably more recent clades than those that enclose the mostly terrestrial early Mesozoic archosaurs. Furthermore, Senter [92] stated that the absence of a septomaxilla in archosaurs is evidence against the presence of a VN system in all members of the clade. However, a septomaxilla is also absent in all extant therian mammals [102], the vast majority of which have a VN system [100]. Thus, the absence of a septomaxilla seems to be an ambiguous osteological correlate for assessing the absence of a VN system in extinct tetrapods. Additional studies and evidence are necessary to assess the presence or absent of a VN system in archosaurs (see also [103]).

If the second interpretation is followed (i.e. the entire impression in front of the olfactory tract belongs to the olfactory bulbs) it implies that the olfactory bulbs of *Tasmaniosaurus triassicus* were unusually large (Table 3), resembling the condition observed in, for example, some baurusuchid crocodyliforms (e.g. *Wargosuchus australis*: [104]). Indeed, the olfactory bulbs of *Tasmaniosaurus triassicus* would be approximately 1.4 times wider the maximum width of the cerebrum, resulting in a proportionally huge olfactory apparatus. Previous authors have indicated that the size of the olfactory bulbs is correlated with olfactory capabilities in vertebrates [105] and with mode of life in, at least, carnivorous mammals [106]. In particular, Gittleman [106] found that in aquatic otters and cats the olfactory bulbs are reduced in comparison with those of fully terrestrial carnivorous mammals and it has been suggested that this reduction in the olfactory system would be related with the diminution of olfactory communication in aquatic environments [107], [108]. Thus, the proportionally large olfactory bulbs of *Tasmaniosaurus triassicus* would undermine previous hypotheses of a semi-aquatic or aquatic mode of life for the species, and possibly also for proterosuchids more broadly [109]–[111], and favour instead a terrestrial mode of life [82], as was recently inferred for *Proterosuchus fergusi* based mostly on palaeohistological evidence [112]. However, the currently ambiguous interpretation of the morphology of the olfactory bulb area of *Tasmaniosaurus triassicus* means that the inferences that can be derived from these soft tissues impressions remain ambiguous.

### Gut Contents

**Figure 19:**
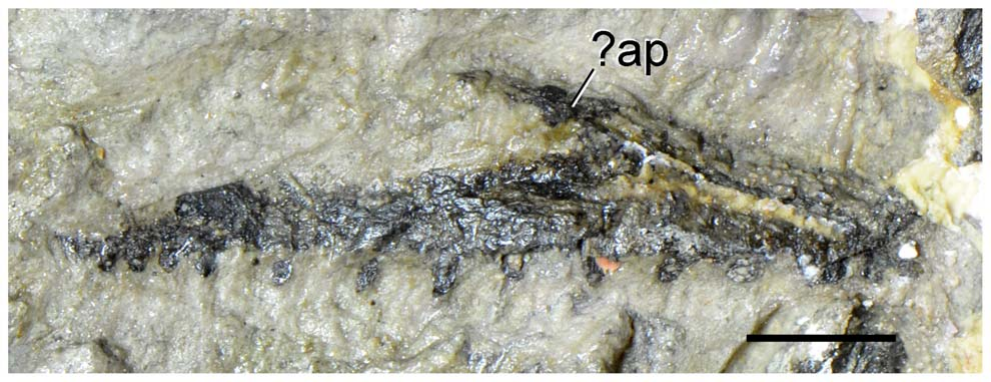
Isolated (?archosauriform) maxilla associated with the type specimen of *Tasmaniosaurus triassicus* (UTGD 54655) in lateral or medial view. Abbreviation: ?ap, possible ascending process. Scale bar equals 5 mm.

Proterosuchids have been historically considered as predatory animals based on dental anatomy and overall skull/mandible morphology (e.g. [110], [111], [113]). In particular, Tatarinov [110] interpreted proterosuchids to be mainly aquatic predators, with larger taxa (e.g. *Proterosuchus fergusi*) feeding upon fish and smaller forms (e.g. *Chasmatosuchus*) feeding on invertebrates. Reig [111] postulated a carnivorous habit for proterosuchids and hypothesized a proterosuchid-dicynodont food web link in Early Triassic assemblages. Subsequently, Sennikov [113] included proterosuchids among the top carnivores of the Late Permian–Early Triassic terrestrial Russian assemblages, feeding upon invertebrates and a high diversity of vertebrates (e.g. dicynodonts, cynodonts, procolophonids, protorosaurs). Thulborn [20] provided the only direct evidence of the diet of proterosuchid archosauriforms when he described supposed gut contents in *Tasmaniosaurus triassicus*. This author reported the presence of a dark grey granular material a few centimetres below the interclavicle in an area in which ribs and gastralia are mixed together. Thulborn [20] suggested that this granular material contained miscellaneous splinters and fragments of bone that may represent the gut contents of *Tasmaniosaurus*. The only fragment of bone identified by this author was a small maxilla (Fig. 19), identified as pertaining to a temnospondyl, which was already originally identified by Camp & Banks [19]. However, some lines of evidence may undermine the interpretation of these bones as gut contents: i) the holotype bones of *Tasmaniosaurus triassicus* are preserved in close association but are not articulated with one another, indicating some degree of preburial transport; ii) there is no recognizable ribcage area and, as a result, the purported gut contents cannot be unambiguously inferred to have been within the animal digestive tract; and iii) the supposed temnospondyl maxilla is preserved in the same kind of matrix as other unambiguous *Tasmaniosaurus triassicus* bones (e.g. interclavicle). Accordingly, the evidence for the presence of gut contents in *Tasmaniosaurus triassicus* is here considered to be ambiguous.

The purported temnospondyl maxilla is not well preserved and is covered with lacquer (Fig. 19). It bears 12 teeth in situ and has a maximum length of 29.5 mm and height of 6.0 mm. Camp & Banks [19] and Thulborn [20] did not provide anatomical evidence supporting the assignment of the maxilla to a temnospondyl. The small maxilla differs from those of temnospondyls in the low tooth count, which is considerably higher in temnospondyls (e.g. [22], [24]), and no character can be identified to support the original identification. On the other hand, the bone resembles in size and overall morphology a partial archosauromorph pterygoid (e.g. the right pterygoid of *Tasmaniosaurus triassicus*). However, this tooth-bearing bone differs from palatal bones in the presence of irregularly spaced teeth, with more closely packed teeth at one end of the bone. The presence of regularly spaced palatal pterygoid teeth in basal archosauromorphs (e.g. *Prolacerta broomi*: BP/1/2675; *Proterosuchus fergusi*: RC 59) undermines the possibility that this bone could be a fragment of the palate of *Tasmaniosaurus triassicus*. The small maxilla seems to have a long and low-angled ascending process that defines the anterior border of a probable antorbital fenestra, resembling the condition observed in archosauriform diapsids [41]. However, the presence of lacquer prevents an assessment of whether or not the area in question is covered with matrix and, as a result, whether or not the supposed border of an antorbital fenestra is an artefact.

If the identification of an ascending process and antorbital fenestra is correct, then the small maxilla can be assigned to the Archosauriformes. The maxilla differs from the almost complete maxilla of the holotype of *Tasmaniosaurus triassicus* in the presence of a proportionately longer anterior process, lower tooth count and proportionately lower height. The maxilla could represent an early juvenile of *Tasmaniosaurus triassicus* or another kind of archosauriform, but the uncertainties in the interpretation of its anatomy means that such a hypothesis must be treated with caution. Accordingly, it seems that the holotype of *Tasmaniosaurus triassicus* was mixed with a possible small archosauriform prior to its burial.
